# MicroRNA-452-5p regulates fibrogenesis via targeting TGF-β/SMAD4 axis in SCN5A-knockdown human cardiac fibroblasts

**DOI:** 10.1016/j.isci.2024.110084

**Published:** 2024-05-23

**Authors:** Iqra Mushtaq, Tsung-Han Hsieh, Yao-Chang Chen, Yu-Hsun Kao, Yi-Jen Chen

**Affiliations:** 1 International Ph.D. Program in Medicine, College of Medicine, Taipei Medical University, Taipei, Taiwan; 2 Joint Biobank, Office of Human Research, Taipei Medical University, Taipei, Taiwan; 3 Department of Biomedical Engineering, National Defense Medical Center, Taipei, Taiwan; 4 Graduate Institute of Clinical Medicine, College of Medicine, Taipei Medical University, Taipei, Taiwan; 5 Department of Medical Education and Research, Wan Fang Hospital, Taipei Medical University, Taipei, Taiwan; 6 Cardiovascular Research Center, Wan Fang Hospital, Taipei Medical University, Taipei, Taiwan; 7 Taipei Heart Institute, Taipei Medical University, Taipei, Taiwan

**Keywords:** Cardiovascular medicine, Molecular biology, Cell biology

## Abstract

The mutated SCN5A gene encoding defective Nav1.5 protein causes arrhythmic ailments and is associated with enhanced cardiac fibrosis. This study investigated whether SCN5A mutation directly affects cardiac fibroblasts and explored how defective SCN5A relates to cardiac fibrosis. SCN5A knockdown (SCN5AKD) human cardiac fibroblasts (HCF) had higher collagen, α-SMA, and fibronectin expressions. Micro-RNA deep sequencing and qPCR analysis revealed the downregulation of miR-452-5p and bioinformatic analysis divulged maladaptive upregulation of transforming growth factor β (TGF-β) signaling in SCN5AKD HCF. Luciferase reporter assays validated miR-452-5p targets SMAD4 in SCN5AKD HCF. Moreover, miR-452-5p mimic transfection in SCN5AKD HCF or AAV9-mediated miR-452-5p delivery in isoproterenol-induced heart failure (HF) rats, resulted in the attenuation of TGF-β signaling and fibrogenesis. The exogenous miR-452-5p significantly improved the poor cardiac function in HF rats. In conclusion, miR-452-5p regulates cardiac fibrosis progression by targeting the TGF-β/SMAD4 axis under the loss of the SCN5A gene.

## Highlights

• Nav1.5 deficiency enhances cardiac fibroblast activity and attenuates miR-452-5p
• MiR-452-5p regulates fibrogenesis by targeting the TGF-β/SMAD4 axis
• AAV miR-452-5p reduces cardiac fibrosis and improves heart failure in rats

## Graphical abstract

**Figure:**
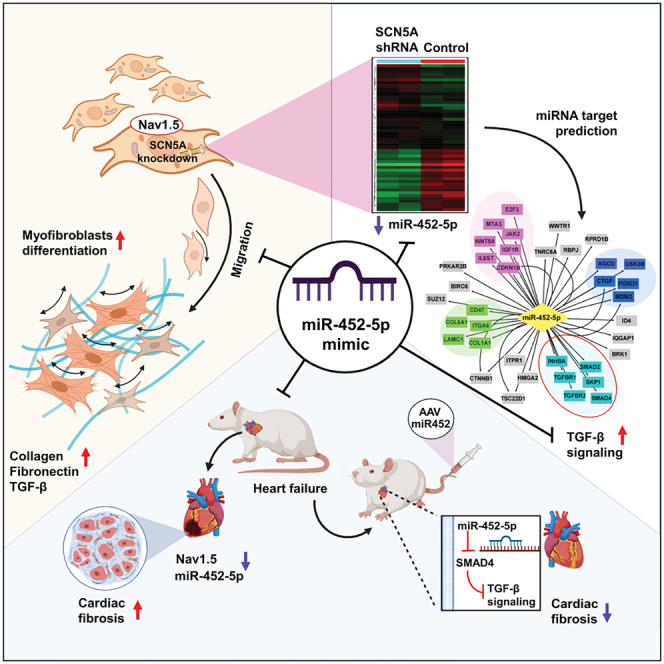

## Introduction

Cardiac arrhythmia affects 33 million people globally and is the prevalent cause of myocardial fibrosis leading to heart failure (HF).^1^ Predominantly, myocardial fibrosis occurs due to abnormalities in ion channels and structural organization. The former contributes to the development of cardiac fibrosis by affecting the initiation and propagation of electrical stimuli, for instance, the reduced *I*_Na_ significantly increased collagen deposition in SCN5A-knockout mice.^2^ SCN5A gene produces α-subunit of the cardiac sodium voltage-gated channel (Nav1.5) arbitrates the rapid Na^+^ influx, which is crucial for the upstroke of action potential and excitation of cardiac cells.^3^^,^^4^ Structural changes in Nav1.5 channel syndicate a spectrum of arrhythmic disorders that lead to sudden cardiac death. Studies reported that the loss of SCN5A gene expression in the human and heterozygous SCN5A mouse models favors the tissue remodeling vulnerability of the heart, and these structural abnormalities are secondary to cardiac sodium channelopathies.^5^^,^^6^^,^^7^^,^^8^ In addition to this, under diminished expression of Nav1.5, distinctive fibrosis has been reported in calcineurin-induced murine cardiac hypertrophic ventricles and Cx43 knockout mice.^9^^,^^10^ Sodium channel dysfunction-dependent onset of cardiac fibrosis and collagen production requires the activation of the transforming growth factor β (TGF-β) pathway. The inhibition of *I*_Na_ triggered the abnormal upregulation of TGF-β receptors,^11^ and SCN5A-knockout mice also exhibited increased expression of TGF-β transcripts.^12^ Under activated TGF-β, fibroblasts differentiate into myofibroblasts which foster extracellular matrix (ECM) secretion. ECM coupled with paracrine factors (TGF-β) and electrical modulators further damages the myocyte-fibroblast’s communication, which then leads to irregular conduction propagation with more signal blockage.^13^ Furthermore, increased collagen release/deposition in ECM increases the myocardium’s proclivity to cardiac dysfunction and HF.^14^^,^^15^^,^^16^ Another study showed that SCN5A decisively contributes to the transdifferentiating of human atrial fibroblasts suggesting the ineluctable involvement of SCN5A mutation in fibroblast activity.^17^ Although the reduced expression of SCN5A plays an essential role in the development of cardiac fibrosis, there is no study on how the defective SCN5A increases the pro-fibrotic TGF-β signaling in human cardiac fibroblasts (HCF).

MicroRNAs (miRNAs) contain 22 nucleotide sequences, crucial as profibrotic or anti-fibrotic by binding to 3ˊ untranslated region (3ˊUTR) of target mRNA.^18^^,^^19^ The plethora of miRNAs has been associated with SCN5A channelopathies and HF, vital regulators in myocardial fibrosis by regulating ECM synthesis and cytokines secretion.^20^ For instance, miR-24 functionally engaged in the regulation of SCN5A expression in patients with HF and miR-210-5p reduces cardiac fibrosis by interfering with TGF-β type I receptor binding in rats.^21^^,^^22^ In addition, miR-452-5p is a putative pathological factor, and significantly downregulated in the cardiac tissues of patients with hypertrophic cardiomyopathy and upregulated in aortic stenosis-induced HF rats.^23^^,^^24^ However, the involvement of miR-452-5p in cardiac fibrosis has not been reported yet, and no research has been done to explore the regulatory function of miR-452-5p in SCN5A-dependent cardiac remodeling.

This study aimed to investigate how the downregulation of SCN5A gene regulates fibrogenesis and clarifies the underlying mechanisms. In the current study, we identified miR-452-5p as a key regulator of SCN5A-induced cardiac fibrosis via miRNA sequencing. We further explored the effect of miR-452-5p on fibroblast differentiation, migration, and the potential molecular mechanism of TGF-β signaling mediation. Our study provides insight into the SCN5A knockdown-induced fibrosis and miR-452-5p as a potential therapeutic target.

## Results

### SCN5A knockdown promotes fibrosis in human cardiac fibroblasts

**Figure 1:**
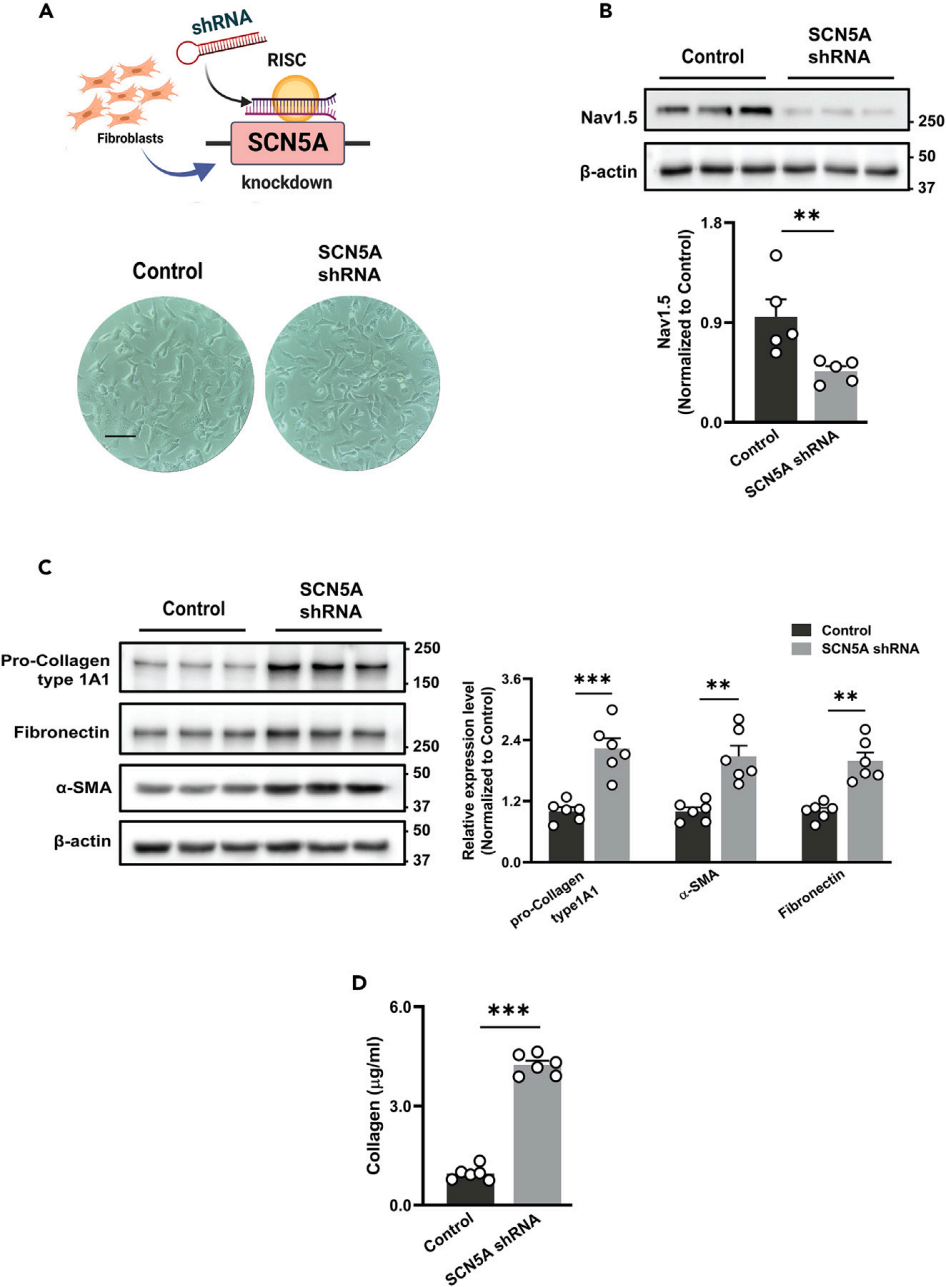
SCN5A knockdown promotes the expression of fibrogenic signaling (A) *Upper panel:* Knockdown of SCN5A gene in human cardiac fibroblasts by targeting SCN5A gene using shRNA lentivirus. *Lower panel*: fibroblast grown after SCN5A gene knockdown and morphology analyzed microscopically, Scale bar 350 μm (representative pictures shown). (B) The protein expression of Nav1.5 after the knockdown of the SCN5A gene in HCF represents a 50% decrease in Nav1.5 protein expression. Data are expressed as mean ± SEM, paired t-test, ∗∗*p* < 0.01, *n* = 5 independent experiments. (C) Representatives immunoblot and quantitative analysis showing the expression of pro-Col1agen 1A1, α-SMA, and fibronectin in SCN5A knockdown and control HCF normalized with the internal control group. Data are expressed as mean ± SEM, paired t-test, ∗∗*p* < 0.01, ∗∗∗*p* < 0.001, *n* = 6 independent experiments. (D) There were higher soluble collagen-type 1 levels measured in a conditioned medium (serum-free) of SCN5A knockdown HCF than that from control cells Data are expressed as mean ± SEM, paired t-test, ∗∗∗*p* < 0.001, *n* = 6 independent experiments. SCN5A shRNA: SCN5A knockdown HCF.

After establishing the SCN5A knockdown cell model, the expression of Nav1.5 was measured via western blot. There was less protein expression of SCN5A by 50% than control HCF without affecting fibroblast morphology (Figures 1A and 1B). To investigate the role of SCN5A knockdown in cardiac fibrosis, the expression level of known pathological cardiac fibrosis markers was checked in SCN5A knockdown and control HCF by western blot. SCN5A knockdown HCF significantly increased the protein expressions of pro-Collagen type 1A1, α-SMA, and fibronectin (Figure 1C). Fibroblasts' ability to synthesize and secrete precursors of fibrillar collagen changes with cardiac remodeling.^25^ As excessive collagen secretion can lead to disproportionate fibrosis, we assessed the difference in collagen secretion capacity between SCN5A knockdown HCF and control HCF. The soluble collagen type-I level in conditioned media was 4-fold higher in SCN5A knockdown HCF compared to control HCF (Figure 1D). These results divulge the crucial commitments of SCN5A knockdown in cardiac fibrosis.

### Differential expression of miR-452-5p, functional enrichment and validation in SCN5A knockdown HCF

**Figure 2:**
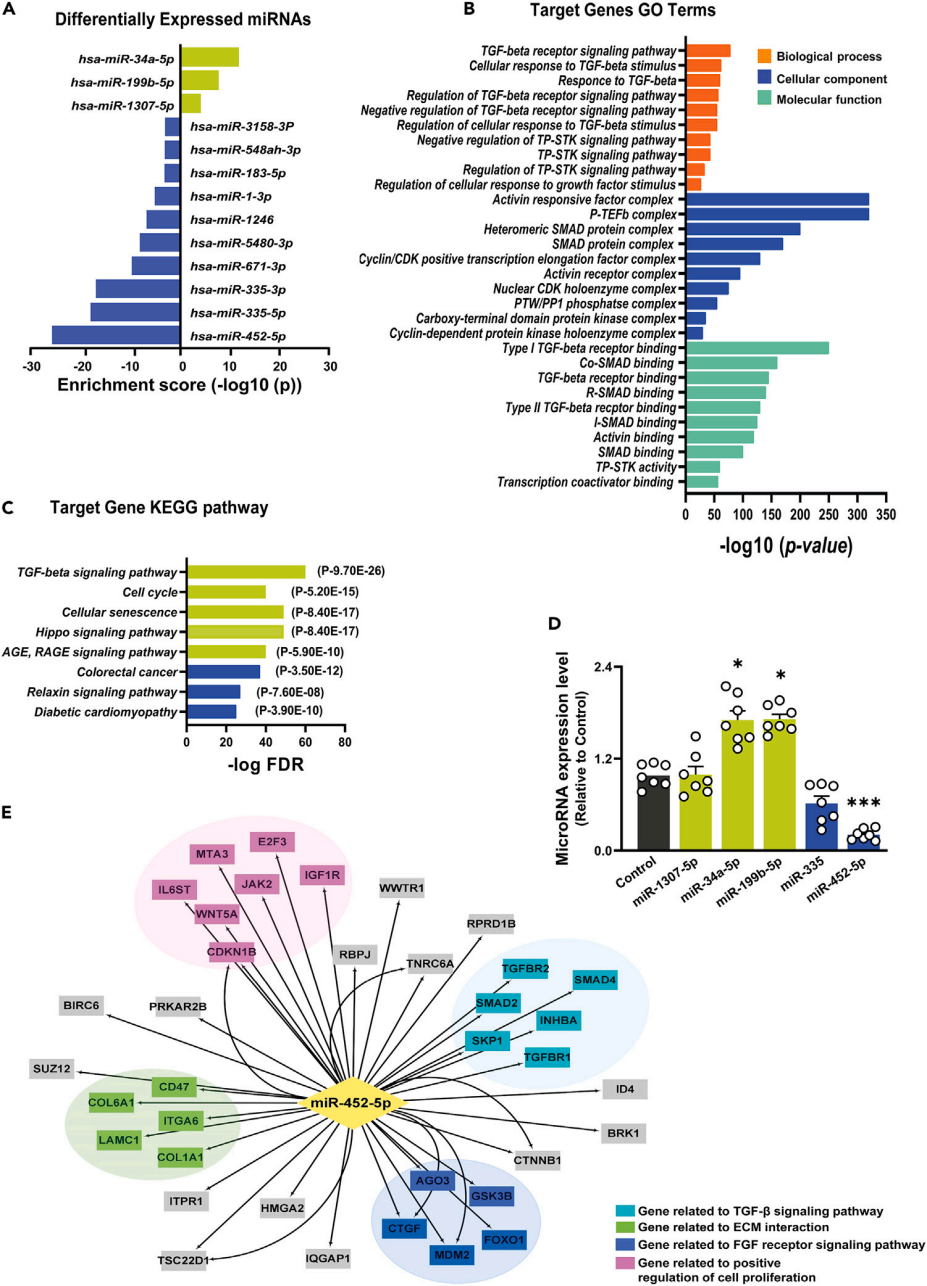
Maladaptive TGF-β signaling is upregulated in SCN5A knockdown HCF (A) Differentially expressed miRs against their enrichment score in SCN5A knockdown HCF. Data plotted against enrichment score, *p* values determined via DESeq2 R package. (B) GO enrichment of differentially expressed miRs target genes (including UP/Downregulated miRs) are presented, top 10 significantly enriched GO term (biological process, cellular component, and molecular function) branches are presented. The GO terms were plotted against fold enrichment values and -log *p*-values (Fisher’s exact test, *p* < 0.05). (C) Top 5 up-regulated (red bars) and top 3 down-regulated (blue bars) significantly enriched pathways in KEGG pathway analysis of differentially expressed genes plotted against -log FDR values. Their corresponding *p*-values calculated by the Fisher’s exact test are mentioned in parentheses in front of the bars. (D) qRT-PCR analysis of micro-RNAs (hsa-miR-1307-5p, hsa-miR-34a-5p, hsa-miR-199b-5p, hsa-miR-335, has-miR-452-5p) identified by small RNA sequencing analysis in SCN5A knockdown and control HCF. Shrek green represents upregulated miRNAs, blue bars represent downregulated miRNAs in SCN5A knockdown HCF, while black bars represent control cells. Data are expressed as mean ± SEM, one-way ANOVA, ∗*p* < 0.05, ∗∗∗*p* < 0.001, *n* = 7 independent biological repeats. SCN5A shRNA: SCN5A knockdown HCF. miR-NTC: miR negative control. (E) Rectangular nodes represent targets mRNA and Diamond nodes represent miRNAs. The network displays mRNAs with the highest predictive score. Edges with arrows indicate the inhibitory effect on targeted mRNA. Light blue nodes show mRNAs involved in the TGF-β signaling pathway. Green nodes illustrate genes related to ECM interaction. Blue and purple nodes show the genes involved in the FGF receptor signaling pathway and positive regulation of cell proliferation respectively. Gray nodes are for genes without specific interpretation. GO: Gene Ontology, KEGG: Kyoto Encyclopedia of Genes and Genome.

We performed hypothesis-free micro-RNA sequencing to identify the possible mechanistic link between the loss of SCN5A and the upregulation of fibrotic genes in HCF. The transcriptomic analysis revealed that a total of 410 microRNAs were expressed in SCN5A knockdown HCF, among which 53 were significantly expressed as shown in the heatmap (Figure S1). A Total of 13 miRNAs were differentially expressed including three miRNAs markedly upregulated and ten were significantly downregulated (Figure 2A). Next, we performed target prediction of upregulated and downregulated miRNAs using multiple tools and identified most common genes predicted by at least two tools (Table S1). These genes were then subjected to GO analysis. (Figure 2B). The KEGG pathway enrichment analysis revealed the most enriched pathways were TGF-β signaling pathway (path:hsa04350), cellular senescence (path:hsa04218), hippo signaling pathway (path:hsa04390), diabetic cardiomyopathy (path:hsa05415), and relaxin signaling pathway (path:hsa04926) (Figure 2C). Furthermore, the differentially expressed miRNAs were verified through stem-loop quantitative real-time PCR. Overall, the top three upregulated and the top two downregulated miRNAs in SCN5A knockdown HCF were selected for validation (Figure 2D). The expression of examined miRNAs showed partial agreement with the RNA-seq data. miR-34a-5p and miR-199b-5p were significantly upregulated, whereas miR-335-5p and miR-452-5p were strikingly lower in SCN5A knockdown HCF, with miR-452-5p being the significantly lowest expressed in SCN5A knockdown HCF. Consequently, we selected miR-452-5p for further functional analysis, and the miRNA-mRNA network was constructed using datasets comprising miRNA-target gene-binding data and miRNA-mRNA interactions using Cytoscape 3.9.1 (Figure 2E). The network analysis unveiled a regulatory abundance of miR-452-5p, with numerous target genes displaying enrichment in TGF-β signaling pathway and genes related to ECM interaction. Specifically, SMAD2, SMAD4, TGF-βRI, and TGF-βRII were notably direct mediators of the TGF-β pathway. Further studies were performed to determine the role of miR-452-5p in SCN5A knockdown-induced fibrosis.

### miR-452-5p attenuated TGF-β signaling in SCN5A knockdown HCF

**Figure 3:**
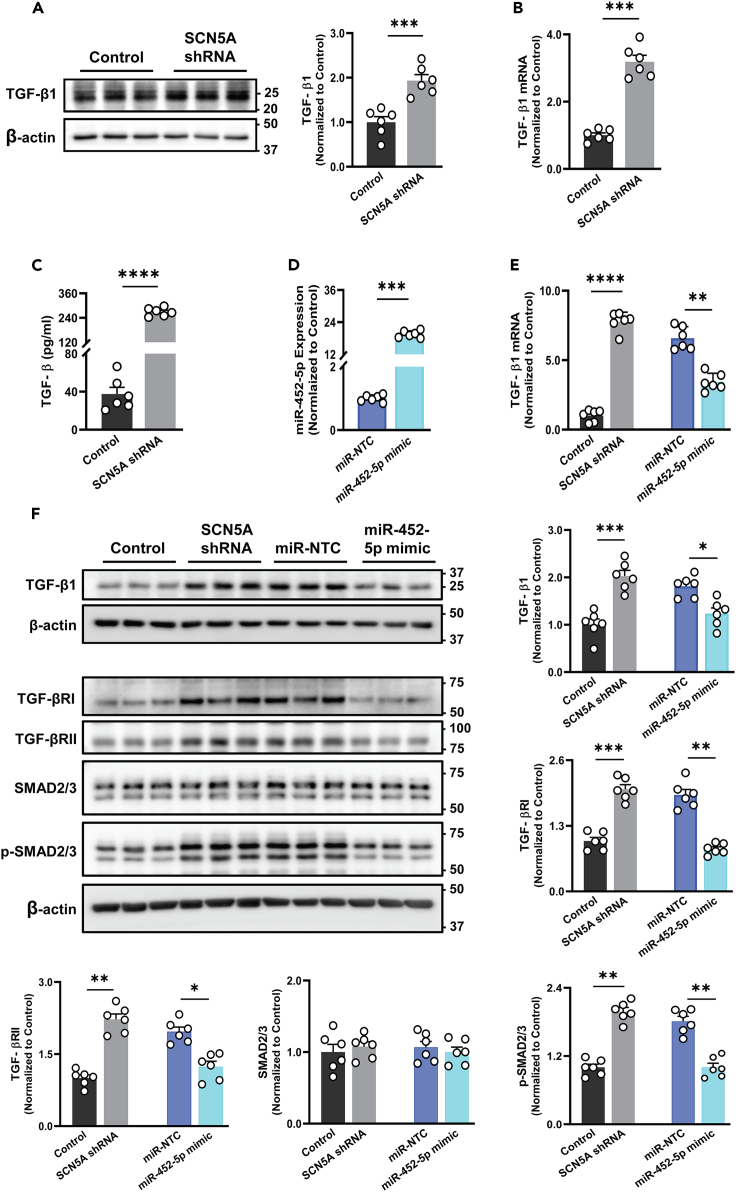
miR-452-5p mimic regulates canonical TGF-β signaling pathway in SCN5A knockdown HCF (A) Representative immunoblot showing the expression of TGF-β1 expression in SCN5A knockdown and control HCF normalized with the internal control group. Data are expressed as mean ± SEM, paired t-test, ∗∗∗*p* < 0.001, *n* = 6 independent experiments. (B) The expression of TGF-β1 mRNA measured via qRT-PCR in SCN5A knockdown and control HCF normalized with the internal control. Data are expressed as mean ± SEM, paired t-test, ∗∗∗*p* < 0.001, *n* = 6 independent experiments. (C) ELISA was used to measure the total secreted TGF-β in a conditioned medium (serum-free) of SCN5A knockdown HCF than that from control cells. Data are expressed as mean ± SEM, paired t-test, ∗∗∗∗*p* < 0.0001, *n* = 6 independent experiments. (D) MiR-452-5p mimic was used to increase the expression of miR-452-5p in SCN5A knockdown HCF and miR-452-5p expression was measured through qRT-PCR. Data are expressed as mean ± SEM, paired t-test, ∗∗∗*p* < 0.001, *n* = 6 independent experiments. (E) TGF-β1 mRNA measured in SCN5A knockdown HCF treated with miR-452-5p and miR-NTC and control HCF via qRT-PCR. Data are expressed as mean ± SEM, paired t-test, ∗∗*p* < 0.01, ∗∗∗∗*p* < 0.0001, *n* = 6 independent experiments. (F) Immunoblots and quantitative analysis of TGF-β1, TGF-βRI, TGF-βRII, total SMAD 2/3, and phospho-SMAD 2/3 in SCN5A knockdown HCF as compared to control and SCN5A knockdown HCF treated without or with miR-NTC and miR-452-5p mimic. *n* = 6 experiments. Data are expressed as mean ± SEM, paired t-test, ∗*p* < 0.05, ∗∗*p* < 0.01, ∗∗∗*p* < 0.001, *n* = 6 independent experiments. SCN5A shRNA: SCN5A knockdown HCF. miR-NTC: miR negative control.

Based on the bioinformatic study, we assessed TGF-β expression in SCN5A knockdown and control HCF. Our western blot and qRT-PCR analysis revealed a significant increase in both TGF-β protein expression and transcripts in SCN5A knockdown HCF compared to the control group (Figures 3A and 3B). Furthermore, the ELISA consolidated the 4-fold increase in secretory form of TGF-β in conditioned media (24 h) from SCN5A knockdown HCF as compared to control (Figure 3C). To validate the functional relevance of miR-452-5p in TGF-β signaling, we treated the SCN5A knockdown HCF with miR-452-5p-mimic (10 nM) or miR negative control (miR-NTC) (10 nM) to increase miR-452-5p expression (Figure 3D), and measured the TGF-βRI, TGF-βRII expression levels because these are TGF-β receptors and their activation leads to phosphorylation of downstream signaling mediators, such as SMAD2 and SMAD3 (canonical TGF-β signaling pathway),^26^ as predicted in miRNA-mRNA network. Interestingly, we found that TGF-β1 transcripts and expression levels of TGF-β1, TGF-βRI, and TGF-βRII, were significantly suppressed by the miR-452-5p mimic than the miR-NTC group which was initially activated in SCN5A knockdown HCF. In line with these results, the phospho-SMAD2/3 expression was noticeably reduced in the miR-452-5p mimic-treated group whereas the total smad2/3 remained unchanged (Figures 3E and 3F). These findings suggest that miR-452-5p actively repressed the TGF-β signaling, which contributed to fibrosis.

### miR-452-5p inhibited fibrosis-related genes, differentiation, and migration of SCN5A knockdown HCF but not restore sodium current (*I*_*Na*_)

**Figure 4:**
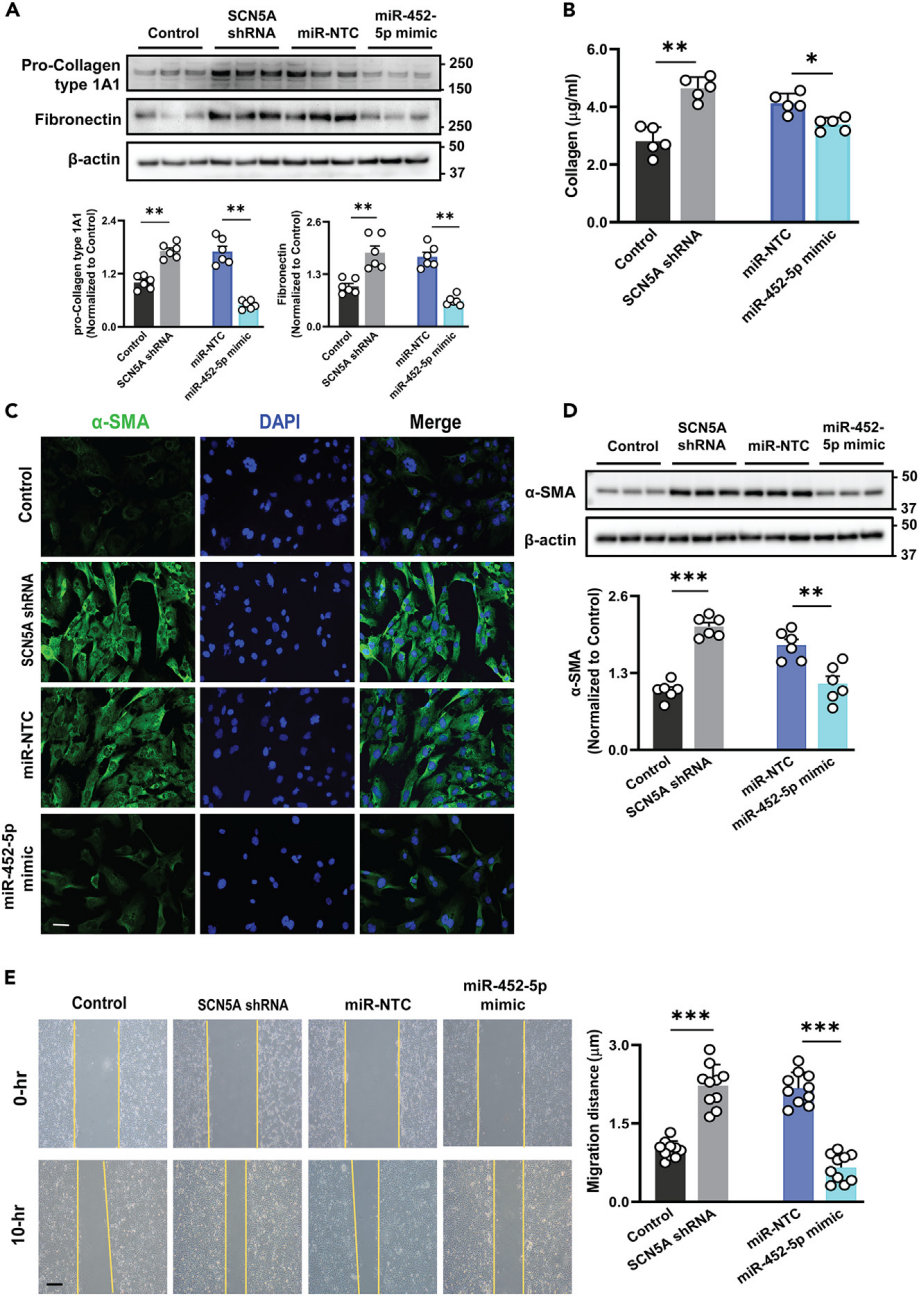
MiR-452-5p mimic restored the fibrogenic phenotype in SCN5A knockdown HCF (A) Immunoblot and quantitative analysis of the expression in protein levels of pro-Collagen type 1A1 and fibronectin in control, and SCN5A knockdown HCF, miR-NTC, and miR-452-5p-mimic groups. Data are expressed as mean ± SEM, paired t-test, ∗∗*p* < 0.01, *n* = 6 independent experiments). (B) Soluble collagen-type1 level measured in conditioned medium from SCN5A knockdown HCF as compared to control and SCN5A knockdown HCF treated without or with miR-NTC and miR-452-5p mimic. Data are expressed as mean ± SEM, paired t-test, ∗*p* < 0.05, ∗∗*p* < 0.01, *n* = 5 independent experiments. (C) Immunohistochemistry shows increased expression of α-SMA in SCN5A knockdown HCF and this expression was significantly reduced upon the treatment of miR-452-5p mimic as compared to both control and miR-NTC groups. α-SMA (green), DAPI (blue). Scale bar: 90 μm. (D) Immunoblots and quantitative analysis of α-SMA in SCN5A knockdown HCF as compared to control and SCN5A knockdown HCF, miR-NTC, and miR-452-5p-mimic groups. Data are expressed as mean ± SEM, paired t-test, ∗∗*p* < 0.01. ∗∗∗*p* < 0.001, *n* = 6 independent experiments. (E) Representative images of migration at 0 h and 10 h post-wounding. Scale bar: 300 μm. Graph showing the cell migration distances (μm) of control, SCN5A knockdown HCF, miR-NTC, and miR-452-5p mimic treated groups. Data are expressed as mean ± SEM, paired t-test, ∗∗∗*p* < 0.001, *n* = 10 independent experiments. SCN5A shRNA: SCN5A knockdown HCF. miR-NTC: miR negative control.

**Figure 5:**
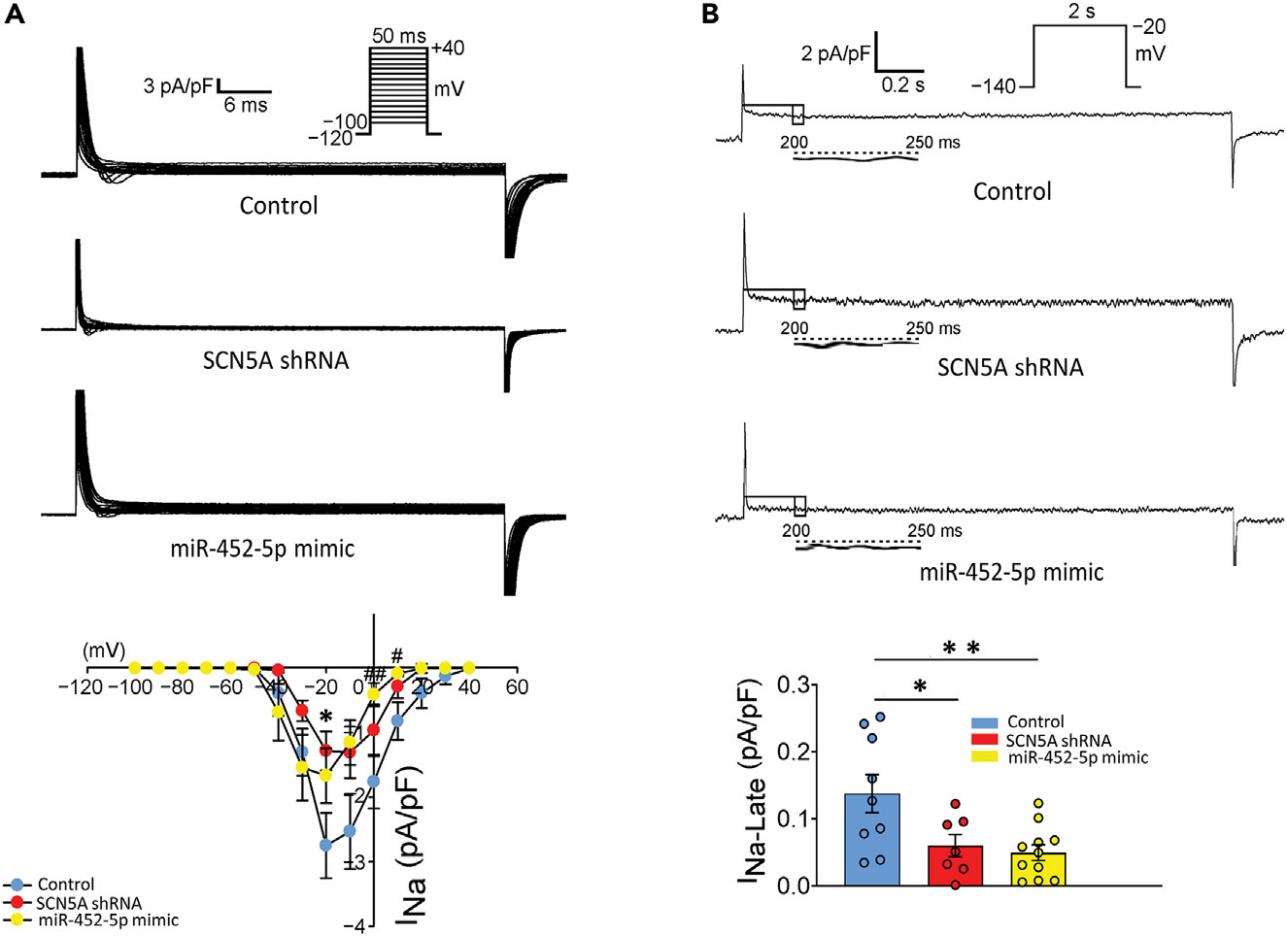
Effect of miR-452-5p on the sodium current (*I*_*Na*_) and late sodium current (*I*_*Na-Late*_*)* in SCN5A knockdown HCF (A) Representative current traces and current-voltage (I–V) relationships of *I*_*Na*_ from control HCF (*n* = 9), SCN5A knockdown HCF (*n* = 10), and SCN5A knockdown HCF transfected with miR-452-5p mimic (*n* = 10). Data are expressed as mean ± SEM, unpaired t-test, ∗*p* < 0.05 Control versus SCN5A knockdown HCF, #*p* < 0.05, ##*p* < 0.01 Control versus miR-452-5p mimic-treated SCN5A knockdown HCF. (B) Representative trace and average data of *I*_*Na-Late*_ from control HCF (*n* = 9), SCN5A knockdown HCF (*n* = 7), and miR-452-5p mimic transfected SCN5A knockdown HCF (*n* = 11). Data are represented mean ± SEM, unpaired t-test, ∗∗*p* < 0.01,∗*p* < 0.05.

To assess the anti-fibrotic effect of miR-452-5p on SCN5A knockdown HCF, we performed immunoblot analysis of SCN5A knockdown HCF treated with miR-452-5p mimic (10 nM) and miR-NTC (10 nM). Our results showed a significant decrease in pro-Collagen type 1A1 and fibronectin levels in the miR-452-mimic treated SCN5A knockdown HCF compared to the miR-NTC group (Figure 4A). In accordance, the concentration of soluble collagen type-I in the medium of miR-452-5p mimic treated SCN5A knockdown HCF reflected the downregulation at the protein level, unlike the miR-NTC group (Figure 4B). Furthermore, our immunofluorescence analyses indicated that SCN5A knockdown dramatically promoted the formation of differentiation-related stress fibers, whereas the miR-452-5p mimic treatment showed a marked decrease in α-SMA fibers as compared to the miR-NTC group consistent with the α-SMA protein expression in miR-452-5p mimic treated SCN5A knockdown HCF (Figures 4C and 4D). Increased myofibroblast differentiation is accompanied by fibroblast cell-migration. To corroborate the role of miR-452-5p in fibroblast migration, we treated SCN5A knockdown HCF with either miR-452-5p mimic or miR-NTC and assessed migration. The wound healing assay showed that SCN5A knockdown enhanced fibroblast migration, while treatment with the miR-452-5p mimic clearly restrained the migration of SCN5A knockdown HCF as compared to the miR-NTC group (Figure 4E). We also investigated whether miR-452-5p could reverse the decreased sodium current (*I*_*Na*_) and late sodium current (*I*_*Na*-Late_) resulting from knockdown of SCN5A gene in HCF. Our findings indicate that miR-452-5p was unable to restore the decreased sodium currents (Figure 5), suggesting that the anti-fibrotic potential of miR-452-5p is exclusively molecular in nature but does not have any effect on *I*_*Na*_ under the loss of SCN5A activity.

### MiR-452-5p directly targets SMAD4 to mitigate fibrogenesis in SCN5A knockdown human cardiac fibroblasts

**Figure 6:**
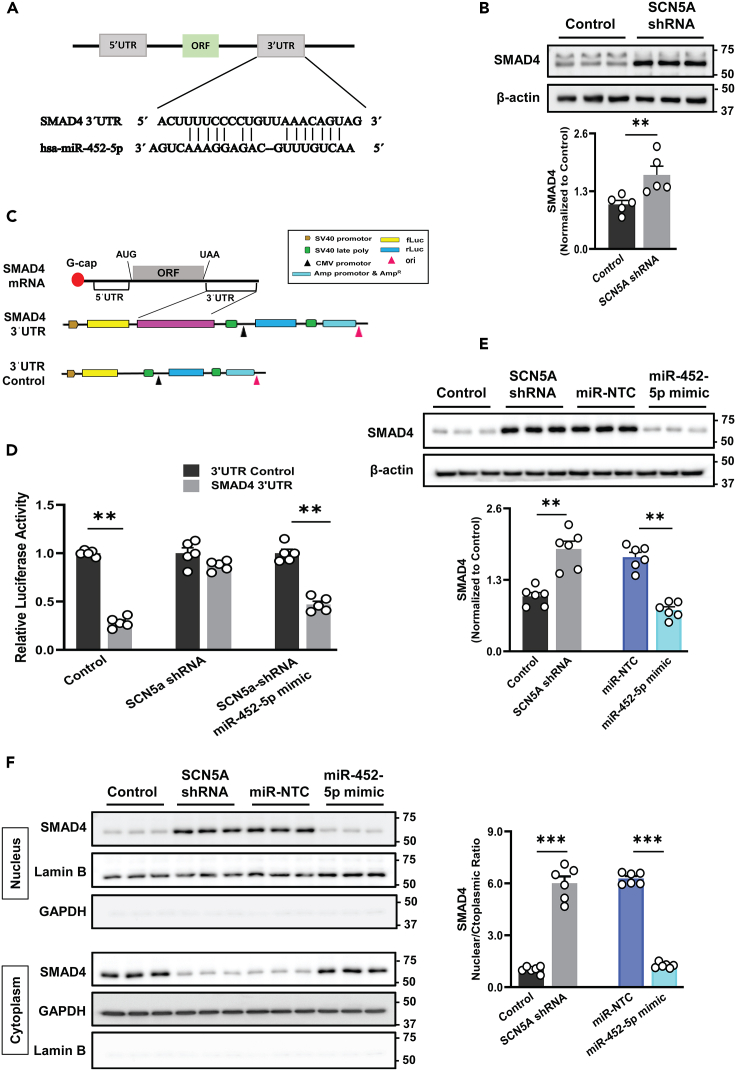
SMAD4: a direct target of miR-452-5p (A) Sequence was predicted from the online database (Target Scan) and complementary targets of 3ˊUTR of human SMAD4 gene with hsa-miR-452-5p. (B) Quantitative analysis and immunoblot representing the protein expression level of SMAD4 in SCN5A knockdown and control HCF. Data are expressed as mean ± SEM, paired t-test, ∗∗*p* < 0.01, *n* = 5 independent experiments. (C) Profile of pEZX-MT06 plasmid for dual-luciferase reporter assay to detect the interaction of miR-452-5p and 3ˊUTR of SMAD4 in SCN5A knockdown vs. control HCF and miR-NTC vs. miR-452-5P mimic in SCN5A knockdown HCF. (D) The luciferase activity was assessed in SCN5A knockdown and control HCF co-transfected without or with miR-NTC and miR-452-5p mimic (10 nM). Data are expressed as mean ± SEM, paired t-test, ∗∗*p* < 0.01, *n* = 5 independent experiments. (E and F) Representative immunoblots and quantitative analysis showing the expression of SMAD4 in whole cell lysate, nuclear, and cytoplasmic fractions from SCN5A knockdown HCF as compared to control and miR-NTC vs. miR-452-5p mimic groups revealed that SMAD4 nuclear translocation blocked by miR-452-5p mimic. Lamin B and GAPDH indicate nuclear and cytoplasmic fractions, respectively. Data are represented as mean ± SEM, paired t-test. ∗∗*p* < 0.01, ∗∗∗*p* < 0.001, n = 5–6 experiments. SCN5A shRNA: SCN5A knockdown HCF. miR-NTC: miR negative control, fLuc: firefly luciferase gene, rLuc: Renilla luciferase gene, CMV-promotor: cytomegalovirus promotor, Amp^R^: ampicillin resistance gene, ori: origin of replication.

To determine the mechanism through which miR-452-5p regulates the TGF-β signaling pathway, analysis using miRanda, predicted pivotal binding sites between miR-452-5p and the SMAD4 mRNA duplex (Figure S2). In addition, the prediction of binding sites in the 3ˊUTR sequence substantiates that among miR-452-5p target genes, 3ˊUTR of SMAD4 contains the most and multiple conserved complementary sites for the seed region of miR-452-5p, corroborating the recruitment of SMAD4 via miR-452-5p (Figure 6A). To verify this hypothesis, we evaluated the expression of SMAD4 in our SCN5A knockdown cell model. The western blot results disclosed that the SMAD4 expression was significantly higher in SCN5A knockdown HCF (Figure 6B). To identify the substantial binding and influence of miR-452-5p on target, we performed 3′UTR reporter assay (Figure 6C). Overexpression of miR-452-5p dramatically amortized the luciferase activity of the SMAD4 3ˊUTR construct and diminished SMAD4 protein level (Figures 6D and 6E), indicating that miR-452-5p may interfere with the nuclear translocation of SMAD4. To confirm this, we performed the nuclear and cytosolic fractionation assay from SCN5A knockdown HCF, miR-452-5p mimic, and miR-NTC treated HCF. As shown in Figure 6F, SMAD4 was translocated into the nucleus of SCN5A knockdown HCF as compared to the control, while the nuclear accumulation of SMAD4 was attenuated in the miR-452-5p mimic group, when compared with the miR-NTC group.

### Treatment with miR-452-5p mimic mitigates fibrosis in isoproterenol-induced HF

**Figure 7:**
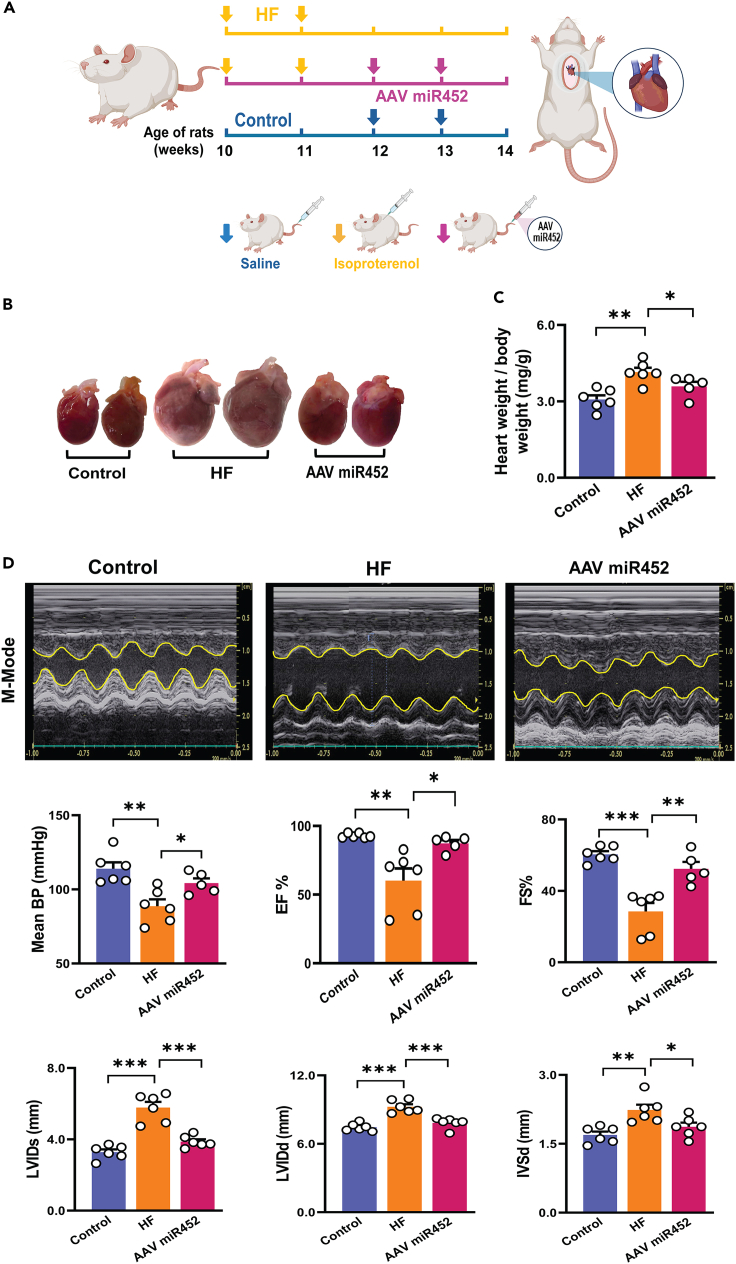
Effect of AAV miR452 on cardiac function in isoproterenol-induced HF (A) Schematic illustration of *in vivo* induction of HF via subcutaneous injection of isoproterenol 100 mg/kg (once a week for 2 weeks) and treatment with AAV miR452 (3.0 x10^10^ genome copies per rat via tail vein, once a week for 2 weeks), control rats received normal saline for 2 weeks. (B) Cardiac pictures from control, HF, and AAV miR452 groups. (C) The ratio of heart to body weight (mg/g) in control, HF, and AAV miR452 groups. Data are presented as mean ± SEM, one-way ANOVA followed by Tukey’s multiple comparison test, ∗*p* < 0.05, ∗∗*p* < 0.01, *n* = 5-6 independent experiments. (D) *Upper Panel*: Representative M-mode echocardiographic images. *Lower Panel*: Averaged data presenting the results of mean blood pressure (BP, mmHg), percentage of ejection fraction (EF%), left ventricle fractional shortening (FS%), left ventricular internal diameter in systole (LVIDs, mm), left ventricular internal diameter in diastole (LVIDd, mm), and intraventricular septal diameter (IVSd, mm) in control, HF, and AAV miR452 groups. Data are represented as mean ± SEM, one-way ANOVA followed by Tukey’s multiple comparison test, ∗*p* < 0.05, ∗∗*p* < 0.01, ∗∗∗*p* < 0.001, n = 5–6 independent experiments.

**Figure 8:**
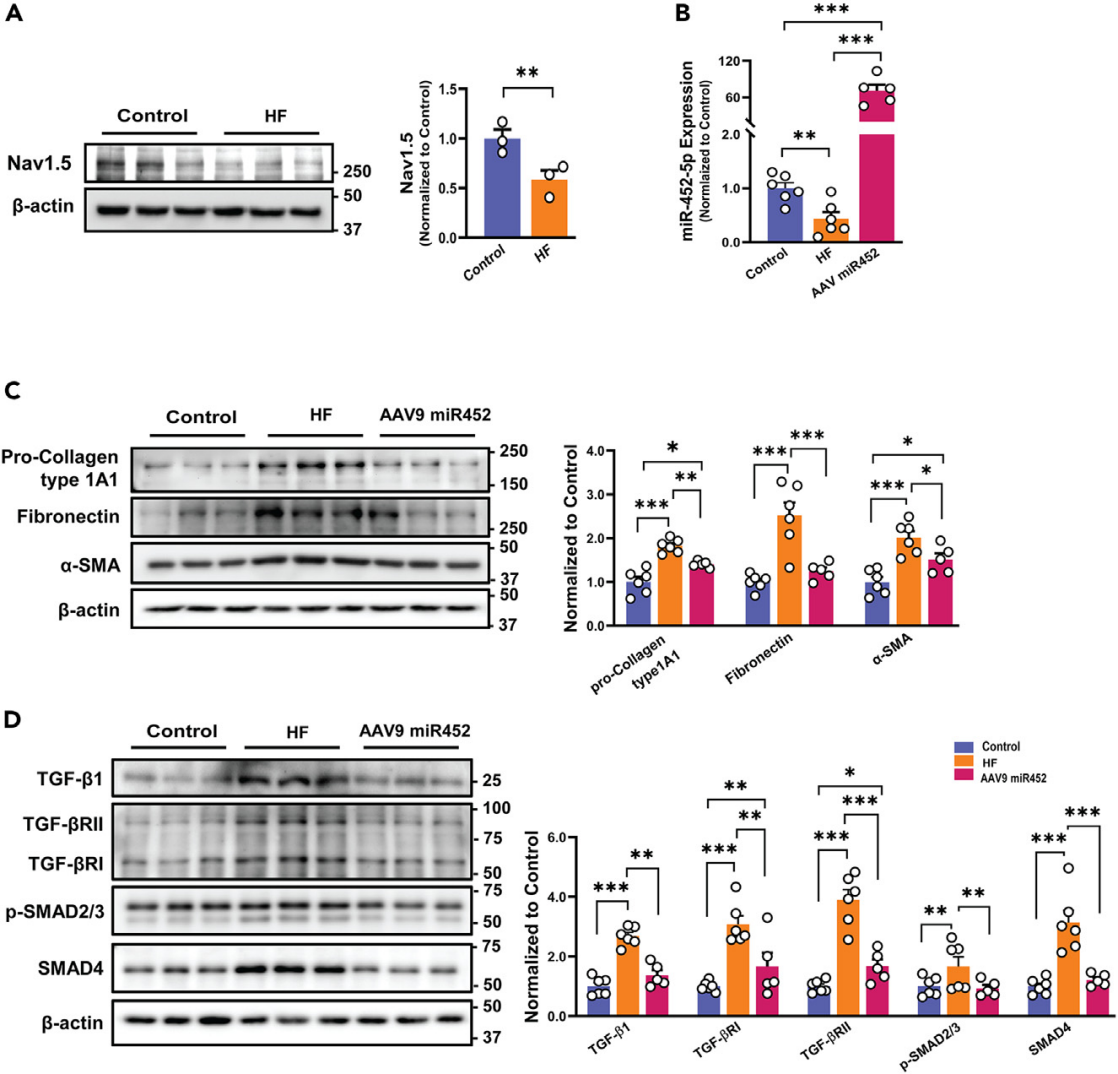
Systemic delivery of AAV miR452 ameliorates fibrosis in isoproterenol-induced HF rats (A) Nav1.5 protein expression in left ventricular tissues of HF and control rats. Data are expressed as mean ± SEM, paired t-test, ∗∗*p* < 0.01, *n* = 3 independent experiments. (B) MiR-452-5p mimic delivery through AAV9 increased the miR-452 expression in left ventricular tissues which was initially reduced after HF development as compared to control, measured via qRT-PCR. Data are expressed as mean ± SEM, one-way ANOVA followed by Tukey’s multiple comparison test, ∗∗∗*p* < 0.001, n = 5–6 independent experiments. (C) Immunoblots and quantitative analysis of pro-collagen type 1a1, fibronectin, α-SMA in LV tissues of HF, and AAV miR452 compared with control. Data are expressed as mean ± SEM, one-way ANOVA followed by Tukey’s multiple comparison test, ∗*p* < 0.05, ∗∗*p* < 0.01, ∗∗∗*p* < 0.001, n = 5–6 independent experiments. (D) Immunoblots and quantitative analysis of TGF-β signaling including TGF-β1, TGF-βRI, RII, *p*-SMAD2/3, SMAD4 in LV tissues of HF (induced by subcutaneous injection of isoproterenol 100 mg/kg once a week for 2 weeks) and HF rats treated with AAV miR452 (3.0 x10^10^ genome copies per rat via tail vein, once a week for 2 weeks) compared with control (received normal saline). Data are represented as mean ± SEM, one-way ANOVA followed by Tukey’s multiple comparison test, ∗*p* < 0.05, ∗∗*p* < 0.01, ∗∗∗*p* < 0.001, n = 5–6 independent experiments.

HF is a common heart disease with enhanced cardiac fibrosis and acquired down-regulation of Nav1.5.^21^^,^^27^^,^^28^^,^^29^ To examine whether miR-452-5p could rescue the fibrogenic phenotype under HF conditions, we generated HF rat model by subcutaneous injection of isoproterenol (100 mg/kg) at the age of 10 weeks. After 2 weeks rats were given the intravenous injection (tail vein) of AAV9-miR-452-5p (3.0 x10^10^ genome copies per rat, once a week for 2 weeks) and saline as negative control once a week. After 2 weeks, rats were sacrificed, and the heart and the serum were collected (Figure 7A). We found that AAV miR452 markedly recovered the fibrotic area in the left ventricular region of the heart as well as the heart weight/body weight ratio (Figures 7B and 7C). Improved cardiac function including restored mean blood pressure, ejection fraction, fraction shortening, left ventricular diameter both in systole and diastole, and thickness of intraventricular septum was observed in AAV miR452 rats compared to HF rats (Figure 7D). As shown in Figure 8A, the expression of Nav1.5 in the ventricular tissues of HF rats were significantly reduced. We also identified the downregulation of miR-452-5p in the HF rats, and it was increased after the delivery of miR-452-5p mimic via AAV9 (Figure 8B). Consistent with *in vitro* findings, over-expression of miR452-5p reversed fibrosis in AAV miR452 group (Figure 8C). Impaired TGF-β signaling in HF was significantly retrieved after treatment with AAV-miR452, SMAD4 expression was also attenuated in AAV9 miR-452 group (Figure 8D). We conducted liver and kidney function tests at the end of the experiments to examine the potential toxicity of the viral vector. There were similar ALT, AST, and BUN in different groups, which implies the non-toxicity of AAV9 (Figure S3). Collectively, these data illustrate the notable significance of *in vivo* administered miR-452 in ameliorating the pathophysiology of the HF animal model.

## Discussion

Cardiac fibrosis provokes pathological changes thereby promoting arrhythmia and HF.^30^ Despite the quintessential advancement in the acknowledgment of cardiac fibrosis and SCN5A channelopathies, the molecular mechanism underlying SCN5A knockdown-induced fibrogenesis remains unclear. Suppression of the SCN5A gene could lead to a defective Na_v_1.5 channel and dropped the peak sodium current (*I*_*Na*_) by altering the gating property of the sodium channel, and conduction is eventually compromised in the heart, accentuating the normal Na^+^ homeostasis.^5^^,^^6^ Concordant with the studies, we found a significant drop in *I*_*Na*_ or late sodium currents (*I*_*Na-late*_) after SCN5A knockdown, quite parallel to the molecular change caused by Nav1.5 deficiency. Studies have reported that SCN5A plays an eloquent role in ventricular fibrosis in old Scn5a+/− mice fibrotic remodeling around the coronary vessel of young Scn5a+/− mice with a higher level of TGF-β.^4^^,^^12^^,^^31^ Another study showed that Nav1.5-E3 antibody directly escalates TGF-β1 synthesis in both cultured HCF and myocytes by blocking Na^+^ channels^32^ Similarly, we found enhanced fibrogenesis with over-expression of pro-collagen type 1A1, fibronectin, and α-SMA in SCN5A knockdown HCF as compared to wild type. Increased α-SMA is a hallmark of mature fibroblasts.^33^^,^^34^^,^^35^ Moreover, the accumulation of collagen in the myocardium is the main culprit leading to cardiac fibrosis and a recent study have shown that this process could accelerate in SCN5A heterozygous knockout mice.^36^ Our immunoblot results for collagen expression are in favor of these studies demonstrating that the SCN5A gene role extends beyond electrical activity but crucial for the maintenance of normal physiological function of the heart.

The upregulation of fibrotic markers (collagen) is accompanied by increased TGF-β1 transcripts and reduced Nav1.5 expression in *Scn5a*^+/−^ mice.^12^ The current study confirms this causal connection between reduced Nav1.5 and upregulated TGF-β in the SCN5A knockdown cell model and left ventricular tissues of the isoproterenol-induced HF rat model. Nav1.5 deficiency activates the TGF-β signaling pathway via its interacting proteins^37^ and contributes to the increase of fibrotic markers turnover. Once, TGF-β1 released it binds to receptor molecules (TGF-βRI & TGF-βRII) with subsequent phosphorylation events further engage in the TGF-β1/SMAD signaling cascade by creating a heterodimeric complex with the transcription factor SMAD4, culminating in the nucleus, causing gene transcription and fibroblast differentiation-induced cardiac fibrosis, and HF.^38^^,^^39^^,^^40^ The current study showed that decreased SCN5A may activate TGF-β/SMAD signaling in knockdown HCF with increased TGF-βI transcripts to establish a positive feedback loop, resulting in cardiac fibrosis.

Based on small RNA deep-sequencing followed by bioinformatic target prediction, we verified that miR-452-5p was downregulated after the knockdown of SCN5A gene further validated through qRT-PCR. Recent studies showed the significant downregulation of miR-452-5p in cardiac tissue of patients with hypertrophic cardiomyopathy correlated with cardiac fibrosis.^23^^,^^33^ The role of miR-452-5p has not been reported in SCN5A knockdown-induced CF. Our data identified the lower expression of miR-452-5p in SCN5A knockdown cell model and the left ventricular tissue of the isoproterenol-induced HF rat model might contribute to the establishment of diseases.

MicroRNAs involvement in the inhibition of the TGF-β/SMAD cascade was extensively reported in mice models with MI, and cardiac hypertrophy^41^^,^^42^ and addressed as a therapeutic strategy for patients with HF.^43^ Our study found that miR-452-5p mimic delivery into SCN5A knockdown HCF resulted in a marked decrease in TGF-β signaling. SCN5A likely facilitates the miR-452-5p expression by interacting with associated proteins and reduced SCN5A/miR-452-5p axis may contribute to TGF-β activity and excessive activation of fibroblasts (increased α-SMA) and could lead to cardiac fibrosis pertinence. Consequently, *in vitro* treatment with miR-452-5p mimic significantly reversed the expressions of α-SMA and collagen in SCN5A knockdown HCF. We also found that miR-452-5p mimic also decreased the migration of SCN5A knockdown HCF. These findings indicated that the downregulation of SCN5A/miR-452-5p is indispensable for TGF-β dependent cardiac fibrosis. In contrast, miR-452-5p mimic does not derepress *I*_*Na*_ and *I*_*Na-late*_ in SCN5A knockdown HCF with and without treatment of miR-452-5p mimic, suggesting that miR-452-5p may not have any effect on retrieving damaged *I*_*Na*_. Moreover, myofibroblast’s transdifferentiation from fibroblasts with enhanced secretory products may cause a shielding effect^17^ to block miR-452-5p action in SCN5A knockdown HCF.

In addition, SCN5A knockdown HCF was also characterized by increased levels of miR-34a-5p as compared to the control. Several studies curated the involvement of miR-34a-5p in cardiovascular diseases, its role in cardiac fibrosis is a bit controversial. Overexpression of miR-34a in rodents' hearts reduces cardiac fibrosis by inhibiting SIRT1 and collagen type 1A1 expression.^44^^,^^45^^,^^46^^,^^47^ In contrast, we found increased pro-Collagen type IA1 in both whole cell lysate and conditioned medium, along with enhanced migration of SCN5A knockdown HCF. Notably, there were no significant alterations observed in the total form of SIRT and phospho-SIRT1 expression levels in SCN5A knockdown HCF (Figure S4). This data emphasizes the cell/tissue-specific role of miR-34a in fibrogenesis, whereas our in-silico analysis does not reveal a substantial association between miR-34a and TGF-β signaling in SCN5A knockdown vs. control HCF. However, additional studies are needed to investigate the role/involvement of miR-34a-5p in SCN5A knockdown-induced cardiac fibrosis.

**Figure 9:**
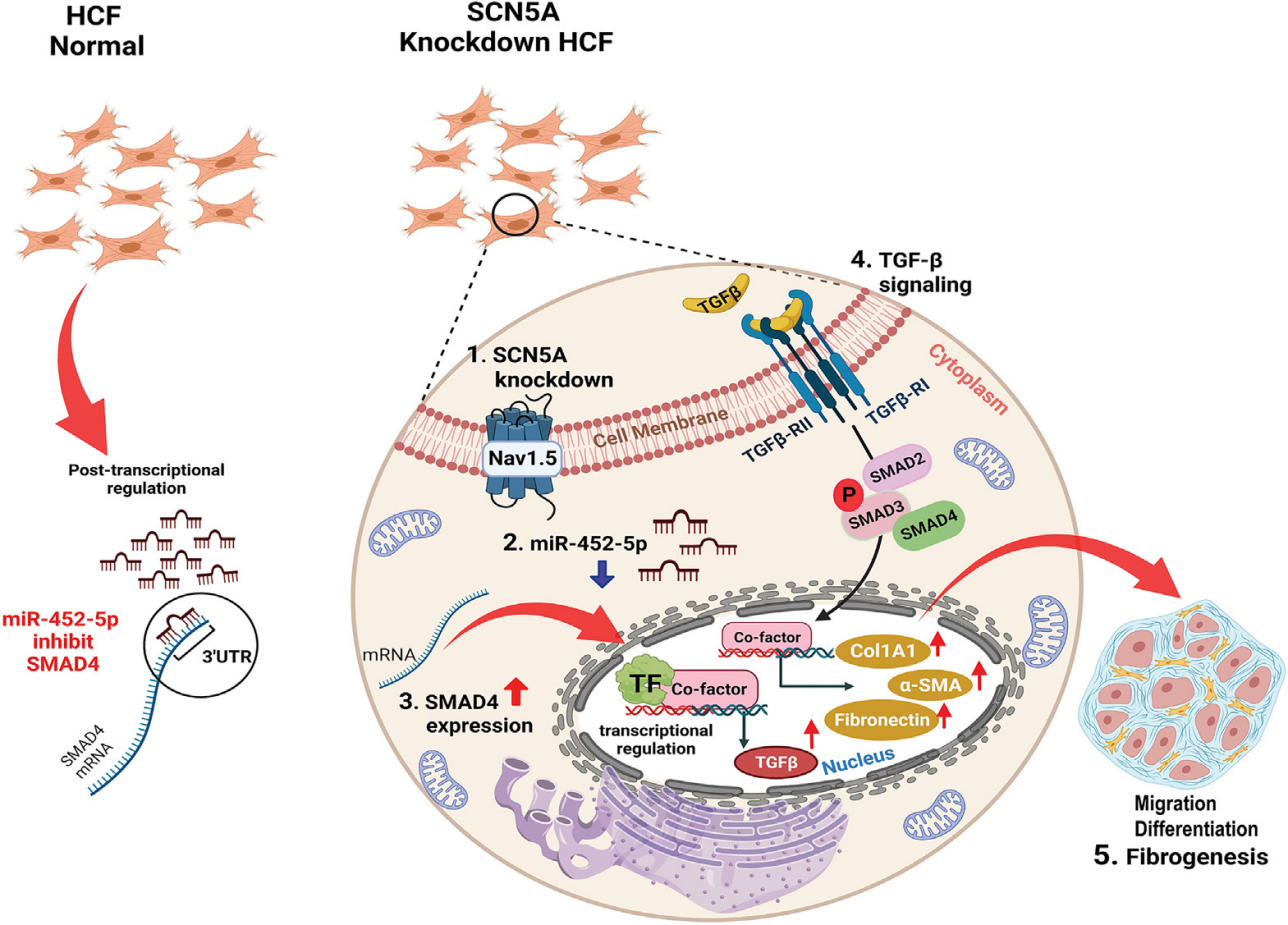
The proposed mechanism of shielding potential of miR-452-5p in cardiac fibroblasts against SCN5A knockdown escalated cardiac fibrogenesis The expression level of miR-452-5p in normal HCF serves to restrain the activity of SMAD4 protein by binding to the 3ˊUTR of SMAD4 mRNA, therefore limiting its activity. However, in the case of SCN5A knockdown, the quantity of miR-452-5p is considerably reduced, impairing its capacity to bind with 3′UTR of SMAD4 mRNA and thereby increasing SMAD4 activity. The increase in SMAD4 activity causes the TGF-β to be overexpressed which activates the canonical TGF-β signaling pathway by increasing the phosphorylation of downstream signal transducers SMAD2 and SMAD3. Following phosphorylation, these form a heterodimer with SMAD4 and translocate into the nucleus, where they increase the expression of fibrogenesis-related genes such as pro-Collagen 1A1, fibronectin, and fibroblasts differentiation by overexpressing α-SMA, and increased cell migration leading to cumulative effect on fibrogenesis in SCN5A knockdown condition.

Mechanistically, we authenticated SMAD4 as a direct target of miR-452-5p in SCN5A knockdown HCF. SMAD4 is a master intracellular mediator of TGF-β pathway,^48^ and crucial for maintaining homeostasis in cardiomyocytes and mouse hearts.^49^ SMAD4 has been reported as one of the targets of miR-34a. Our comparative analysis based on binding score (curated from miRDB, miRanda, miRWalk, and DIANA-microT) showed that miR-452-5p exhibited a stronger affinity for SMAD4 mRNA in comparative analysis (Table S2). A previous report indicates that miR-452-5p abrogate posttranscriptional SMAD4 activity in human renal cell carcinoma cell lines.^50^ By reporter assay, our study confirmed the direct binding of miR-452-5p on the 3ˊUTR of SMAD4 in SCN5A knockdown HCF. Hence, miR-452-5p negatively regulates SMAD4 expression, intruding its nuclear localization (Figure 9). MiR-452-5p mimic restrained the positive loop of TGF-β/SMAD signaling leading to cardiac fibrosis. Moreover, studies reported a strong association between the decrease of SCN5A expression and worse HF outcomes in human, aged Scn5a^+/−^ mice, and canine chronic HF models.^21^^,^^28^^,^^51^ In accordance, our *in vivo* experiments showed lowered expressions of Nav1.5 and miR-452-5p in the isoproterenol-induced HF rats, suggesting that loss of SCN5A/miR-452-5p axis may actively promote cardiac fibrosis in HF. Besides, curative delivery of miR-452-5p by AAV9 can rescue against cardiac fibrosis. Taken together, overexpression through either miR-452 mimic transfection or AAV9-miR-452-5p injection could reverse SCN5A deficiency-mediated cardiac remodeling in both SCN5A knockdown HCF and isoproterenol induced HF. This suggests that targeting miR-452-5p/SMAD4 could offer promising therapeutic avenues for managing cardiac fibrosis in HF patients with SCN5A deficiency.

In conclusion, miR-452-5p has been identified as a regulator of cardiac fibrosis development. This miRNA influences the phenotypic switch and migration in SCN5A knockdown HCF, as well as modulates the TGF-β signaling pathway. It achieves this regulation by targeting SMAD4, thereby suppressing TGF-β signaling, and systemic delivery to animals is sufficient to reduce SCN5A knockdown-mediated fibrosis. These findings clarify the significant involvement of miR-452-5p/SMAD4 in the development of cardiac fibrosis and propose a promising target for therapeutic perspective to manage the enhanced cardiac fibrosis associated with SCN5A knockdown.

### Limitations of the study

Our present work demonstrated that SCN5A knockdown mediated cardiac fibrosis induced by downregulation of miR-452-5p, and its normal expression is indispensable for the regulation of SMAD4 verified through the reporter assay. However, how the knockdown of the SCN5A gene could downregulate miR-452-5p expression in HCF is still unclear and may require further investigations.

Our study indicates an absence of AAV9 toxicity. However, this might be attributed to the short duration of AAV9 exposure. It is possible that prolonged exposure could have different outcomes, although the potential off-target effect of AAV9 could not be entirely excluded in the current study. These possibilities are intriguing subjects for future studies to understand the complex and coordinated regulation of miR-452-5p in SCN5A-dependent cardiac remodeling. Additionally, further studies are mandatory to explore the potential of circulating miR-452-5p as a reliable and sensitive biomarker for HF in clinical scenarios. Extensive clinical studies and functional analyses are warranted to validate and expand upon these initial findings.

## STAR★Methods

### Key resources table

**Table:** 

REAGENT or RESOURCE	SOURCE	IDENTIFIER
**Antibodies**		

Anti-Nav1.5	Alomone	Cat# ASC-005; RRID:AB_2040001
Anti-Pro-Collagen type 1A1	Santa Cruz	Cat# sc-293182; RRID:AB_2797597
Anti-α-SMA	Abcam	Cat# ab7817; RRID:AB_262054
Anti-Fibronectin	Millipore	Cat# MAB1940; RRID:AB_94436
Anti-TGF-β1	Cell Signaling	Cat# 3711; RRID:AB_2063354
Anti-TGF-beta receptor I	Sigma	Cat# SAB 1300113; RRID: AB_10609530
Anti-TGF beta receptor II	Cell Signaling	Cat# 3713; RRID: AB_2240491
Anti-SMAD2/3	Cell Signaling	Cat# 8685; RRID:AB_10889933
Anti-P-SMAD2/3	Cell Signaling	Cat# 8828; RRID:AB_2631089
Anti-SMAD4	Santa Cruz	Cat# sc-7966; RRID:AB_627905
Anti-Sirt1	Abcam	Cat# ab32441; RRID:AB_777937
Anti-P-Sirt1	Cell Signaling	Cat# 2314; RRID:AB_561516
Anti-beta-Actin	Santa Cruz	Cat# sc-47778; RRID:AB_626632
Anti-LaminB	Santa Cruz	Cat# sc-6216; RRID:AB_648156
Anti-GAPDH	Mbl	Cat# M171-7; RRID:AB_10699462
Anti-mouse ab	R&D System	Cat# HAF007; RRID:AB_357234
Anti-rabbit ab	R&D System	Cat# HAF008; RRID:AB_357235
Goat Anti-Mouse IgG H&L (Alexa Fluor 488)	Abcam	Cat# ab150117; RRID:AB_2688012

**Bacterial and virus strains**		

AAV9	AAV Core Facility, Academia Sinica	CA-1521

**Chemicals, peptides, and recombinant proteins**		

FM-2	Science Cell	Cat#2331
Lipofectamine RNAiMAX Transfection reagent	Thermofisher	Cat#13778150
Lipofectamine 3000 Transfection reagent	Thermofisher	Cat#L3000001
Opti-MEM	Thermofisher	Cat#31985-062
Trypsin EDTA	Sartorius	Cat#03-051-1B
Trizol	Invitrogen	Cat#15596018
Trizol LS reagent	Life Technology	Cat#10296010
Bromo-3-chloropropane	Sigma	Cat#B9673
Propanol	Sigma	Cat#19516
PVDF membrane	Millipore	Cat#IPVH00010
Acrylamide	BioRad	Cat#1610158
Paraformaldehyde	Sigma	Cat#P6148
Triton™ X-100	Sigma	Cat#18787
BSA	Bioshop	Cat#ALB001
PCR Master Mix	Thermofisher	Cat#4304437
Ammonium peroxydisulfate (APS)	Sigma	Cat#1003351259
Sodium dodecyl sulfate (SDS) powder	Sigma	Cat#102283911
TEMED (N, N, N’, N’-Tetramethylethylenediamine)	BioRad	Cat#161-0801
ECL Select Western Blot Detection Reagent	Cytiva	Cat#RPN2235
M-PERTM Mammalian Protein Extraction Reagent	Thermofisher	Cat#78501
Protease and phosphatase inhibitor cocktail	Abcam	Cat#ab201120
SuperScript III reverse transcriptase	Invitrogen	Cat#18080093
Isoproterenol	Millipore	Cat#I003551522

**Critical commercial assays**		

Qubit™ Protein Assay Kits	Thermofisher	Cat#Q33212
Sircol Soluble Collagen Assay	Biocolor	Cat#S1111
Human/Mouse/Rat/Porcine/Canine TGF-beta1 Quantitative ELISA	R&D System	Cat#DB100C
NE-PER Nuclear and Cytoplasmic Extraction reagents	Thermofisher	Cat#78835
TaqMan^R^ microRNA Assay Kit	Applied Biosystem	Cat#4427976
TaqMan^R^ microRNA RT kit	Applied Biosystem	Cat#4366596
ReverTra Ace qPCR RT kit	Toyobo	Cat#FSQ-301
Luc-PairTM Duo-Luciferase Assay Kit 2.0	GeneCopeia	Cat#LF001

**Deposited data**		

Raw miRNA-Seq data	This paper	GEO: GSE261598
Bio Project I. D	This Paper	PRJNA1075457

**Experimental models: Cell lines**		

Human Cardiac Fibroblasts	Innoprot	Cat# P10453-IM; RRID:CVCL_YJ37

**Experimental models: Organisms/strains**		

Male Wistar rats	BioLASCO	N/A

**Oligonucleotides**		

Primers for qPCR (See Table S3)	This paper	N/A
miRNA 3’UTR target Clone	GeneCopeia	Cat#CmiT000001-MT06
miRNA 3’UTR target clone (a, b) SMAD4	GeneCopeia	Cat#HmiT102069(a, b)-MT06

**Recombinant DNA**		

AAV9-miR-452	AAV Core Facility, Academia Sinica	AB-026
Plasmid: pEZX-MT06	GeneCopeia	Cat# HmiT102069-MT06

**Software and algorithms**		

AlphaEaseFC	Alpha Innotech	Version: 7.0.1
MyCurveFit package	My Assays	https://mycurvefit.com/
GraphPad Prism 8	GraphPad Software	Version: 8.0.2
Image J	NIH	Version: 2.1.0; RRID:SCR_003070
miRDB	https://mirdb.org	RRID:SCR_010848
MiRanda	https://microrna.org	Version: 3.3.a; RRID:SCR_017496
Target Scan Human v.8.0	https://www.targetscan.org	RRID:SCR_010845
miRWalk v.3	http://mirwalk.umm.uni-heidelberg.de	RRID:SCR_016509
Cytoscape 3.9.1	https://cytoscape.org	RRID:SCR_003032
R Studio	https://cran.r-project.org	Version: 2022.02.3

**Other**		

small RNA seq	Illumina	N/A

### Resource availability

#### Lead contact

• Prof. Yu-Hsun Kao. Graduate Institute of Clinical Medicine, College of Medicine, Taipei Medical University, No. 250, Wu-Hsing Street, Taipei 11031, Taiwan. Email: yuhsunkao@gmail.com.

#### Materials availability

This study did not generate any unique reagent.

#### Data and code availability

• The small RNA sequencing data has been submitted to GEO under the accession number GSE261598 and is publicly available as of the date of publication. Raw reads and sample information accessible through Project I.D. PRJNA1075457.
• The current study did not create any original code or command.
• Any additional information related to the current study will be provided on lead contact on reasonable grounds.

### Experimental model and study participant details

#### HF rat model

Male Wistar rats of 8-week-old were purchased from BioLASCO Taiwan Co., Ltd and housed in the animal facility of Taipei Medical University under standard conditions (20-22°C temperature, 12:12 light and dark cycle, normal rat chow feed) for two weeks. At the age of 10 weeks, HF was induced by administering a subcutaneous injection of 100 mg/kg of isoproterenol (Sigma, St. Louis, Mo., USA) dissolved in normal saline once a week for 2 weeks. Echocardiography is performed each week to asses the HF development. Rats receiving normal saline were designated as the control group. All the experiments followed the guidelines approved by the *Institutional Animal Care and Use Committee of Taipei Medical University and* received approval from the Local Animal Ethics Review Board (LAC-2024-0133).

#### SCN5A knockdown cell model

The immortalized human cardiac fibroblasts (HCF, male, Innoprot, Derio, Spain) were cultured in fibroblast medium 2 (FM-2) with 10% heat-inactivated fetal bovine serum and antibiotics (penicillin 100 U/mL and streptomycin 100 μg/mL) in a 37°C incubator with 5% CO_2_. To study the functional relevance of Nav1.5 deficiency, and establish the SCN5A knockdown cell model, the SCN5A gene was knocked down using the lentivirus shRNA system (Academia Sinica, Taipei, Taiwan). 293T cells were transfected using three plasmids: envelop plasmid (pMD2.G), packaging plasmid (pCMVΔR8.91), and shRNA plasmid (pLKO.1 shRNA) with 1:9:10 proportion. The supernatant collected after 48 h post-transfection, comprising viral particles, was filtered using a 0.45 μm filter. Fibroblasts were then treated for 24 h with polybrene and virus medium, selected with 10 μg/ml puromycin for 7 days, and maintained in media encompassing 1 μg/ml puromycin. The cells were subsequently cultured and expanded through subculturing, and these (<6 passage number) cells was utilized for the experiments.

#### Construction of AAV miR452 vector

For the construction of adeno-associated virus (AAV) vectors expressing rno-miR-452, a 685 bp region encompassing the rno-miR-452 precursor (85 nts) and 300 nts of its flanking genomic sequence on both sides to ensure proper processing of miRNA precursor into mature miRNA was initially cloned into the pUC57 vector (GenScript, NJ, USA). Subsequently, the inserted sequence, including the AgeI and HindIII restriction sites, was PCR-amplified using the forward primer pmiR-452 F (5’-CCCGCGGCCGATCC*ACCGGT*ATTCCTCTTTTCCACATTGTTTTCAGTTTC-3’) and the reverse primer pmiR-452 R (5’-CGACGGTATCGAT*AAGCTT*TATTACTGTAGCCATTGCACCTGAG -3’). The resulting amplification products were cloned into an AgeI and HindIII-linearized dsAAV vector using the In-Fusion HD Cloning Plus kit (Takara Bio, Inc., Shiga, Japan) and used in further experiments.

### Method details

#### Immunoblot analysis

SCN5A knockdown HCF and control HCF grown in a 6-well plate were lysed by utilizing the M-PER™ Mammalian Protein Extraction Reagent (Thermo Scientific, Cat. #78501) with protease and phosphatase inhibitor cocktail (EDTA free) (Abcam, ab201120). Protein concentration was measured with Qubit™ Protein Assay Kits (Thermo Scientific, Ref #Q33212). Equal amounts of protein were used for SDS-PAGE and transferred onto PVDF membranes (Millipore, IPVH00010). Next, the membranes were incubated with primary antibodies targeting pro-Collagen type 1A1, SMAD4, LaminB, and β-actin (Santa Cruz, sc-293182, sc-7966, sc6216, and sc4778, respectively); α-SMA (Abcam, ab7817); Nav1.5 (Almone Labs, AC-005); Fibronectin (Millipore, MAB1940); TGF-beta receptor I (Sigma, SAB1300113); TGF-β1, TGF-beta receptor II, phospho-SMAD2/3 (Cell signaling, 3711, 11888, and 8828, respectively); as well as GAPDH (Mbl, M171). Following primary antibody incubation, membranes were exposed to appropriate secondary antibodies as listed in key resources table. Bound antibodies were identified using ECL Select western blotting detection reagent (Cytiva, Amersham, UK) and evaluated via AlphaEaseFC (Alpha Innotech, San Leandro, USA). To ensure comparable protein loading, the targeted bands were normalized to β-actin then to control bands.

#### Soluble collagen measurement and ELISA for TGF-β

SCN5A knockdown and control HCF were cultured in a 96-well plate for collagen measurement and in a 6-well plate for TGF-β estimation. After cell growth up to 60% confluency, the culture medium was replaced with serum-free medium (100 μl) and further incubated for 24 h in serum-free media for both assays.

For secretory collagen measurement, conditioned medium was collected after 24 h and collagen concentration was measured using Sircol Soluble Collagen assay (Biocolor, County Antrim, UK), according to manual instructions with sample absorbance measured at 556 nm.

For secretory TGF-β estimation, after 24 h of incubation, the concentrations of TGF-β in the conditioned medium were measured using ELISA (DB100C, R&D Systems, BioTechne, Minneapolis, MN, USA). To activate the latent TGF-β1, the supernatants were acidified with HCl (1N, 20 μl) and incubated at 37°C for 10 minutes. Subsequently, the acidified samples were neutralized (1.2 N NaOH/0.5M HEPES, 20 μl) and the concentration was measured at 450 nm, based on the kit’s manual. A 4PL curve-fit was generated using the MyCurveFit package.

#### Small RNA deep sequencing

Total RNA was collected from SCN5A knockdown and control HCF using Trizol reagent (Invitrogen) according to the kit’s guidelines. Thermo Fisher Scientific's NanoDrop was employed to gauge RNA concentration. A bioanalyzer and Qubit were used to evaluate the quantity and quality of RNA. RNA-sequencing (RNA-seq) was used to carry out expression profiling. 1 ug of total RNA was subjected to directional RNA adapter ligation. The ligation product was reverse transcribed, and PCR amplified to produce a cDNA library appropriate for high-throughput sequencing by NEBNext® Multiplex Small RNA Library Prep Set for Illumina, Ipswich, MA, USA). The libraries then performed size selection using Pippin Prep™ to acquire the small RNA fraction. All next-generation sequencing experiments were performed on an Illumina NovaSeq 6000 by single-end sequencing with a 50 bp read length. Unique sequences were allied with known miRNAs and quantified as absolute expression values. The raw sequencing reads included in FASTQ files were submitted to adaptor trimming, mirbase alignment, and feature counts were determined using the Rsubread algorithm after the sequencing was complete. The FASTQC (version 0.11.8) tool also produced a quality report. Prior to statistical analysis, expression data were normalized to correct the technical biases, and log2 was transformed. Different miRNA expressions were determined using the DESeq2 R package. Sorting was done on DEGs that had statistical significance (p<0.05 and FDR<0.05).

#### Target prediction, GO, and pathway analysis

In silico analysis was executed to ascertain the validated gene candidates for differentially expressed miRNAs in SCN5A-knockdown and control HCF. The analysis employed specific prediction criteria, including a prediction score threshold of 60, miRNAs positioned at nucleotides 5-10 in 3ˊUTR of mRNAs, and only biologically conserved target sites (across species) within the seed region of the miRNAs were considered.^52^ The possible gene targets of miRNAs were determined through the intersection of the prediction made by four algorithms namely miRDB (https://mirdb.org/), miRanda (https://microrna.org), Target Scan Human v.8.0 (https://www.targetscan.org) and miRWalk v.3 (http://mirwalk.umm.uni-heidelberg.de).^53^ Subsequent functional analysis of these identified genes, utilizing Gene Ontology (GO) and Kyoto Encyclopedia of Genes and Genomes (KEGG) pathway analyses was performed utilizing ShinyGO v0.741 (http://bioinformatics.sdstate.edu) and DAVID (https://david.ncifcrf.gov/tools.jsp), and significance determined by the fisher exact test.^54^^,^^55^ For network analysis, selected results were further visualized with Cystoscape 3.9.1 (https://cytoscape.org).^56^

#### Stem-loop qRT-PCR and qPCR

Total RNA was collected from SCN5A knockdown and control HCF. Micro-RNAs were reverse-transcribed by utilizing the TaqMan® microRNA RT kit (Applied Biosystems, USA, #4366596) and the corresponding stem-loop primers (TaqMan® microRNA assay kit, #4427975). A stem loop-based RT-qPCR procedure was executed for the identification of miRNAs, as formerly explained.^57^ Briefly, total RNA was diluted to a concentration of 2 ng/μl, with 5 μl added to 7 μl of reaction mix (0.15 μl of 100 mM dNTPs, 1 μl of 50 U/μl Multiscribe™ reverse transcriptase, 1.5 μl of 10X reverse transcription buffer, 0.19 μl of 20 U/μl RNase inhibitor, and 4.16 μl of nuclease-free water), and 3 μl of 5X RT specific primer (Table S3) to a final volume of 15 μl. Reverse transcription was performed following standard cycling temperatures (incubated for 30 minutes at 16°C, followed by 30 minutes at 42°C, with a 5-minute stop reaction at 85°C, and then held at 4°C). For PCR amplification, 0.67 μl of cDNA template was added to a PCR reaction mix (0.50 μl of TaqMan™ small RNA Assay 20X, 5μl of PCR master mix (#4304437), and 3.84 μl of nuclease-free water) with a final volume of 10 μl. The cycling conditions included enzyme activation at 95°C for 10 minutes, followed by 40 cycles of denaturation at 95°C for 15 seconds and annealing/extension at 60°C for 60 seconds. For TGF-β gene expression analysis, total RNA was procured from control HCF, SCN5A knockdown HCF, and SCN5A knockdown HCF treated with miR-NTC and miR-452-5p mimic. Reverse transcription and qPCR were performed using the ReverTra Ace™ qPCR-RT kit according to the manual instructions. Subsequently, mRNA expression of TGF-β1 was quantified using the ABI PRISM7300 system (Applied Biosystems). All RT-PCR reactions were performed in triplicates The relative changes in the transcript level of the target gene were determined by analyzing the threshold cycle (Ct) value and normalizing it to the specific Ct value of glyceraldehyde-3-phosphate dehydrogenase recorded in the relevant SCN5A knockdown HCF samples, followed by that of control HCF.

#### *In vitro* miR-452-5p mimic transfection

SCN5A knockdown HCF was treated with hsa-miR-452-5p mimic AACUGUUUGCAGAGGAAACUGA, mirVana® miRNA mimic, Assay ID: MC12509, Thermo and miRNA negative control (miR-NTC, mirVana™ miRNA Mimic, Negative Control #1, Thermo) utilizing lipofectamine 3000 transfection reagent (Invitrogen, Thermo Fisher Scientific, L3000001) according to manual’s briefings. Briefly, SCN5A knockdown HCF was cultured in a complete medium. First, 1 × 10^5^ cells were cultured in a 6-well plate for 24 h before transfection. Next, the culture was transfected with miR-452-5p mimic (10 nM) and miR-NTC (10 nM) utilizing Lipofectamine 3000, dissolved in Opti-MEM (Life Technologies Corporation, USA), and the culture was supplied with complete medium (2 ml). The transfection reagent was added for 8 h, then washed off, and incubated in a complete medium until 48 h. The serum-free medium was used for TGF-β1 and soluble collagen type 1 expression analysis. Following the transfection period, the medium and cells were collected for further *in vitro* analysis.

#### Immunofluorescence and migration analysis

SCN5A knockdown HCF, control, and SCN5A knockdown HCF treated with miR-NTC (10 nM) and miR-452-5p mimic (10 nM) grown on glass cover slip, 80% confluent cells were then washed with 1X PBS, fixed with 4% paraformaldehyde in PBS (15 minutes), permeabilized with 1% Triton (5 minutes), and blocked with 3% BSA (1 hr). Cells were subjected to incubation with primary antibody for α-SMA (ab7817) and secondary antibody conjugated with AlexaFluor and DAPI for overnight and 2 hr respectively. Next, coverslips were mounted on glass slides and imaged using Revolve R4 microscope (ECHO). The scratch assay was performed on the cultured SCN5A knockdown HCF, control, and SCN5A knockdown HCF treated with miR-NTC (10 nM) and miR-452-5p mimic (10 nM) by scraping the confluent monolayer of cell culture utilizing the P200 pipette, then imaged at 0 hr and 8 hr by SPOT software (Diagnostic Instruments, Sterling Heights, MI). Gap length (in μm) covered by migrated cells analyzed by ImageJ software.

#### Duo-luciferase reporter assay

To construct a PCR-specific SMAD4 plasmid (SMAD4 3' UTR), 3' UTR sequences were procured from public-domain gene sequence databases and inserted, amplified, and cloned into pEZX-MT06 (GeneCopeia, Madison, WI, USA). The control vector without the 3' UTR of SMAD4 (CmiT000001-MT06, GeneCopoeia) and the vector containing 3' UTR of SMAD4 with predicted target sequence of miR-452-5p (HmiT102069a-MT06, HmiT102069b-MT06, GeneCopoeia) were transfected into control HCF, SCN5A knockdown HCF, miR-NTC, and miR-452-5p mimic groups (24-well plate; 5x10^4^ cells/well) using lipofectamine 3000 dissolved in Opti-MEM (Life Technologies Corporation, USA), nurtured in complete medium for 8 h then washed off, and incubated in a complete medium for 48 h. Based on the kit's manual, the luciferase activity was assessed by using a Luc-PairTM Duo-Luciferase Assay Kit 2.0 (GeneCopeia, Madison, WI, USA).

#### Nuclear and cytoplasmic extraction

Nuclear and cytoplasmic fractions were extracted from SCN5A knockdown and control HCF, which were precultured for 24 h and then transfected with miR-NTC (10 nM) and miR-452-5p mimic (10 nM). The separation of cytoplasmic and nuclear fractions was performed using the NE-PER nuclear and Cytoplasmic extraction reagents (Thermo fisher, Waltham, MA, 78835) according to the manufacturer's instructions. Briefly, cell cultures were trypsinized, centrifuged at 500 x g for 3 minutes, and the supernatant was discarded. The pellet was then resuspended in cytoplasmic extraction reagent I (CERI) and cytoplasmic extraction reagent II (CERII) and incubated for 10 and 5 minutes, respectively. To separate the cytoplasmic extract, the mixture was centrifuged at 16,000 x g for 5 minutes, and the supernatant was collected. Subsequently, the pallet containing the nuclear extract was resuspended in ice-cold nuclear-extraction reagent (NER), further centrifuged at 16000 x g for 10 minutes, and the supernatant containing nuclear extract was separated. The samples were stored at -80°C for further analysis.

#### Measurement of sodium current

Control and SCN5A knockdown HCF were cultured in 6-well plate (1 × 10^5)^, after 24 h the SCN5A knockdown HCF was treated with miR-452-5p mimic (10 nM). The transfection reagent was added for 8 h, then washed off, and incubated in a complete medium until 48 h. Next, the supernatant was removed, and cells were trypsinized for sodium current recordings. Whole-cell patch-clamp recordings were performed at 35±1°C using an Axopatch 1D patch clamp amplifier. Voltage command pulses were generated by a personal computer equipped with an analog-digital converter (Digidata 1200, Molecular Devices) using pCLAMP software v8.0 (Molecular Devices). The internal solution for *I*_*Na*_ was supplemented with (mM): 35 NaCl, 105 CsF, 0.1 EGTA, and 10 HEPES. The pH was adjusted at 7.4 using CsOH. The bath solution contained (mM) NaCl 150, KCl 2, MgCl_2_ 1, CaCl_2_ 1.5, HEPES 10, and glucose 10 at pH of 7.4 using NaOH. *I*_*Na*_ was generated by clamping the cell membrane from a holding potential of -120 mV to a potential ranging from -100 mV to +40 mV for 50 milliseconds in 10 mV increments with 3-second stimulus intervals. *I*_*Na-Late*_ was recorded at room temperature (22–24°C) with an external solution containing (in mmol/L): 140 NaCl, 5 CsCl, 2.0 MgCl2, 1.8 CaCl2, 5 HEPES, 5 glucose, and 0.002 nicardipine. The amplitude of _INa-Late_ at a voltage of -20mV was measured as the mean current amplitude between 200 ms and 250 ms after the membrane was depolarized by a 2000-ms pulse from -140 to -20 mV.

#### HF model and AAV9-mediated delivery of miR-452-5p

Male Wistar rats were randomly divided into three groups i.e., control, HF, and HF treated with AAV9-miR-452-5p (AAV miR452). HF development was assessed one week after the completion of isoproterenol administration using echocardiography. Subsequently, the HF and miR452 groups were categorized upon confirmation of HF. The rats from group AAV9-miR-452-5p were tail vein-injected with single-stranded AAV9-miR-452-5p (3.0 x10^10^ genome copies per rat) using the hydrodynamic transfection method. Briefly, a large volume of solution (volume (ml)=total dose(gc)/conc. (gc/ml)) containing AAV vector, dissolved in normal saline, was rapidly injected into the bloodstream which generates a transient increase in hydrostatic pressure, further facilitates the uptake of the genetic material by target organ.^58^^,^^59^^,^^60^ Rats were anesthetized using isoflurane with 5% induction and 2% maintenance, then AAV9-miR452-5p dissolved in saline was rapidly injected into a lateral tail vein while holding the base of the tail vein between the middle and index fingers. Control rats were injected with an equal amount of saline, so it could serve as control for both HF and AAV miR452 groups. The echocardiography was performed on each group after saline, isoproterenol, and AAV9-miR-452-5p injections following the same anesthesia conditions mentioned before. Then, control, HF, and AAV miR452 (after 2 weeks of post-treatment, 14-week age) rats were euthanized, and blood and cross-sectional tissue pieces from the left ventricle were rapidly frozen in the liquid nitrogen for further protein and gene expression analysis.

### Quantification and statistical analysis

All quantitative data are expressed as the mean ± standard error of the mean (SEM) and mentioned in the relevant figure legends. R software (https://cran.r-project.org) was utilized for miRNA data analysis in conjunction, with Fisherˊs exact test for enrichment analysis. The D’Agostino-Pearson or Kolmogorov-Smirnov test was applied to establish whether the data pursued a Gaussian distribution, and the paired t-test was used to establish statistical significance between SCN5A knockdown and control HCF. A nonparametric Mann-Whitney test was used to compare two groups that failed the normality test. One-way Analysis of variance with Tukey’s post hoc test was performed to determine statistical significance between more than one treatment groups. Prism 8 (www.graphpad.com) software was utilized for statistical analysis. P-values <0.05 were deemed as significant differences.
