# Supplementary material for: MicroRNA-452-5p regulates fibrogenesis via targeting TGF-β/SMAD4 axis in SCN5A-knockdown human cardiac fibroblasts
Source: iScience. 2024 May 23;27(6):110084. doi: 10.1016/j.isci.2024.110084 (PMC11179076; doi:10.1016/j.isci.2024.110084)
Supplement: Document S1. Figures S1–S4 and Tables S1–S3 [file mmc1.pdf]

**iScience, Volume *27***

## Supplemental information

# MicroRNA-452-5p regulates fibrogenesis via targeting TGF-β/SMAD4 axis in SCN5A-knockdown human cardiac fibroblasts

**Iqra Mushtaq, Tsung-Han Hsieh, Yao-Chang Chen, Yu-Hsun Kao, and Yi-Jen Chen**

**Table S1. List of significantly expressed miRNAs, targeted signaling, and genes in control versus SCN5A knockdown human cardiac fibroblasts, related to Figure 2.**

| miRNA | Targeted signaling | p-value | Targeted genes |
|---|---|---|---|
| **Up-regulated miRNAs enriched signalings** | | | |
| **miR-34a-5p** | Cell cycle (hsa04110) | 1.42E-05 | HDAC1, E2F1, CDK1, TP53, MYC |
| | Adherens junction (hsa04520) | 3.59E-06 | STAT3, IL6R, TIMP1, MAPK1, IL6, VEGFA, EIF4E2, EP300, EGFR, HIF1A, MAP2K2, NFKB1, STAT3, |
| **has-miR-199b-5p** | ECM-receptor interaction (hsa04512) | 1.39E-26 | LAMB2, CD44, COL5A1, FN1, THBS1, ITGB8 |
| | Hippo signaling pathway (hsa04390) | 2.50E-03 | MYC, CCND2, CTGF, YAP1 |
| **has-miR-1307-5p** | HIF-1 signaling pathway (hsa04066) | 2.68E-03 | TF, MAP2K2, LDHA, CYBB, HK1, MKNK2 |
| **Down-regulated miRNAs enriched signalings** | | | |
| **has-miR-3158-3p** | TGF-beta signaling pathway (hsa04350) | 7.39E-05 | PPP2R1B, SMAD4, THBS1 |
| | Lysine degradation (hsa00310) | 1.32E-04 | KMT2B, KMT2A, KMT2D, OGDH |
| | Colorectal cancer (hsa05210) | 1.67E-03 | APPL1, APC |
| **has-Mir-548ah-3p** | TGF-beta signaling pathway (hsa04350) | 4.93E-03 | SMURF2, TGFBR1, SMAD6, SMAD4, SMAD7, BMP7, SP1, FST, SMAD5 |
| | Cell cycle (hsa04110) | 1.02E-02 | GSK3B, CCNA2, ORC2, HDAC2, YWHAZ, MDM2, EP300, MAD2L1, ATR |
| | Colorectal cancer (hsa05210) | 1.40E-03 | PIK3CB, WNT5A, AR, RUNX1 |
| **has-miR-183-5p** | Hippo signaling pathway (hsa04390) | 1.64E-06 | TCF7L2, YAP1, LLGL1, CDH1, ACTB |
| | Adrenergic signaling in cardiomyocytes (hsa04261) | 1.59E-02 | ATF2, PPP2CA, ATP2B4, PRKACB |
| **has-miR-1-3p** | Glycosphingolipid biosynthesis - lacto and neolacto series (hsa00601) | 8.16E-12 | FUT3, FUT9, GJA1 |
| | Arrhythmogenic right ventricular cardiomyopathy (ARVC) (hsa05412) | 1.31E-04 | GJA1, UST, ACTN2 |
| | Colorectal cancer (hsa05210) | 2.79E-02 | TCF7L2, MAPK3, MAPK1, PIK3R3, CCND1 |
| **has-miR-1246** | Cell cycle (hsa04110) | 2.55E-03 | BAX, CDK6, TP53, CCNG2, CCNE1, CASP5, BCL |
| | N-Glycan biosynthesis (hsa00510) | 6.97E-03 | MAN1A1, MAN1A2 |

| | | | |
|---|---|---|---|
| **has-miR-5480-3p** | TGF-beta signaling pathway (hsa04350) | 1.71E-04 | SMAD2, SMAD3, PPP2R1B |
| | Hippo signaling pathway (hsa04390) | 2.79E-03 | YAP1, YWHAH, AMOT, LATS1, BMP2, DLG2 |
| **has-miR-671-3p** | NF-kappa B signaling pathway (hsa04064) | 2.58E-03 | TRAF2, INEB2, GSG2, UBA3 |
| | Regulation of actin cytoskeleton (hsa04810) | 1.28E-03 | FGF11, ARHGAP35, PIP5K1A |
| **hsa-miR-335-3p** | Cell cycle (hsa04110) | 7.02E-08 | THBS1, CUL2, CDKN1B, NOTCH2, PA2G4, CDK6, TP53INP1 |
| | TGF-beta signaling pathway (hsa04350) | 7.96E-04 | ADAM9, COL3A1, USP15, COL1A2, PARP1, FNTA, SERPINE1 |
| **has-miR-335-5p** | ECM-receptor interaction (hsa04512) | 2.03E-10 | COL3A1, ITGA2, COL1A1, SPP1, THBS3, HCPG2, CD36, LAMB1 |
| | Arrhythmogenic right ventricular cardiomyopathy (ARVC) (hsa05412) | 2.46E-02 | RYR2, ITGB5, JUP |
| | Hippo signaling pathway (hsa04390) | 1.64E-02 | WNT10B, ACTB, CCND2, MYC |
| **miR-452-5p** | TGF-beta signaling pathway (hsa04350) | 1.02E-08 | INHBA, SMAD2, SMAD4, TGFBR1, TGFBR2, SKP1 |
| | ECM-receptor interaction (hsa04512) | 2.21E-06 | ITGA11, ITGA6, COL1A1, COL6A1, DAG1, FN1, CD47 |

**Table S2. Comparative analysis of miR-452-5p and miR-34a-5p targeting SMAD4: nucleotide pairing and prediction scores, related to Figure 6.**

| miRNA | Target | N pairing (nucleotides) | Score | Source Tool |
|---|---|---|---|---|
| MiR-452-5p | SMAD4 | 8 | 80 | miRDB https://mirdb.org/ |
| | SMAD4 | 7 | 170 | miRanda https://microrna.org |
| | SMAD4 | 16 | 1.0 | miRWalk http://mirwalk.umm.uni-heidelberg.de/ |
| | SMAD4 | 10 | 89 | DianaMicroT https://dianalab.e-ce.uth.gr/ |
| miR-34a-5p | SMAD4 | 7 | 76 | miRDB www.https://mirdb.org/ |
| | SMAD4 | 7 | 152 | miRanda https://microrna.org |
| | n/a | n/a | n/a | miRWalk http://mirwalk.umm.uni-heidelberg.de/ |
| | SMAD4 | 9 | 82 | DianaMicroT https://dianalab.e-ce.uth.gr/ |

**Table S3. List of primers utilized in the current study, related to STAR Methods.**

| Gene | Gene Bank Accession number | Primer Names | Sequence (5´–3´) | Annealing temp °C | Product Size (bp) |
|---|---|---|---|---|---|
| TGFB1 | NM_000660 | Forward | TTGATGTCACCGGAGTTGTG | 60 | 112 |
| | | Reverse | TCCACTTGCAGTGTGTTATCC | | |
| GAPDH | NM_002046 | Forward | CCATGTTCGTCATGGGTGTGAACCA | 60 | 251 |
| | | Reverse | GCCAGTAGAGGCAGGGATGATGTTC | | |
| **miRNAs qRT-PCR primers** | | | | | |
| hsa-miR1307-5p | MIMAT0022727 | Mature | UCGACCGGACCUCGACCGGCU | | 77 |
| | | Forward | TACCAATCTCGACCGGACCT | 60.03 | |
| | | Reverse | CTATCTACCACGACCGACGC | 60.04 | |
| hsa-miR-34a-5p | MIMAT0000255 | Mature | UGGCAGUGUCUUAGCUGGUUGU | | 75 |
| | | Forward | TGGCAGTGTCTTAGCTGGTTGTT | 58.84 | |
| | | Reverse | GCAGCACTTCTAGGGCAGTAT | 59.86 | |
| hsa-miR-199b-5p | MIMAT0000263 | Mature | CCCAGUGUUUAGACUAUCUGUUC | | 97 |
| | | Forward | CTAACCCAGCCCAGCCTAAC | 60.32 | |
| | | Reverse | AGTGCAGGGTCCGAGGT | 60.11 | |
| hsa-miR-335 | MI0000816 | Mature | UCAAGAGCAAUAACGAAAAAUGU | | 81 |
| | | forward | TTTTGAGCGGGGGTCAAGAG | 60.25 | |
| | | reverse | AATGAGAGGAGGTCAGGAGC | 58.51 | |
| hsa-miR-452-5p | MIMAT0001635 | Mature | AACUGUUUGCAGAGGAAACUGA | | 76 |
| | | Forward | AGCACTTACAACTGTTTGCAGAG | 59.69 | |
| | | Reverse | GCACTTACTTCTTTGCAGATGAGA | 59.31 | |
| **miR-452-5p mimic and 3'UTR target cloned sequences** | | | | | |
| miR-452-5p-mimic | NIMAT0039350 | Mature | AACUGUUUGCAGAGGAAACUGA | 60 | |
| | MI0010399 | Stem-loop sequence | AGUUUCCUCUGCAAACAGUUTT | 60 | |

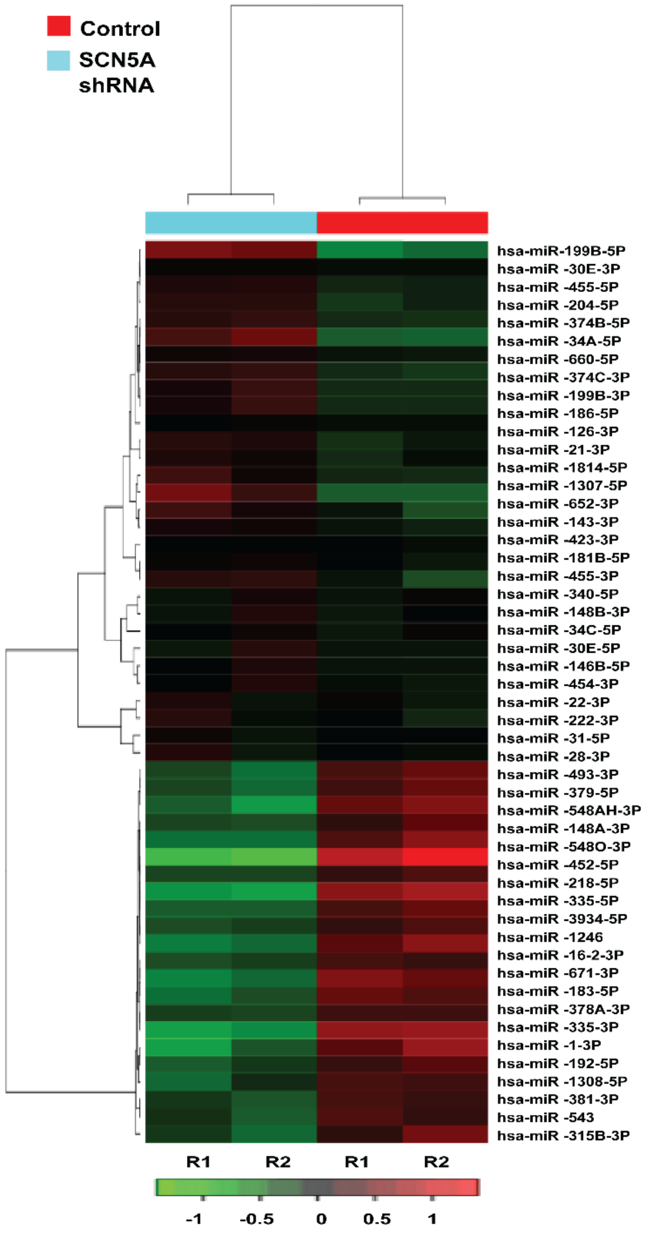


**Figure S1. Differentially expressed miRs, related to Figure 2.**

Transcriptome analysis by RNA-sequencing of SCN5A knockdown and control HCF. DESeq2 R package, n=2 independent biological repeats. Heatmap of hierarchical clustering showing fifty-three significantly expressed miRs in SCN5A knockdown cells as compared to control HCF. The colour code in the heatmap is linear with green as the lowest and red as the highest expression.

<table>
<tr><th>ID</th><th>Duplex structure</th><th>Position</th><th>Score</th><th>MFE</th></tr>
<tr><td>1</td><td>miRNA 3' agucAAAGGAGAC-GUUUGUCAa 5'<br>||||| ||| :|||||||<br>Target 5' tactTTTCCCCTGTTAAACAGTa 3'</td><td>736 - 758</td><td>170.00</td><td>-14.90</td></tr>
<tr><td>2</td><td>miRNA 3' agucaAAGGA-GACGUUUGUCAa 5'<br>||||| |||::||||||<br>Target 5' taccaTTCCTCCTGTGAACAGTg 3'</td><td>3069 - 3091</td><td>157.00</td><td>-16.40</td></tr>
<tr><td>3</td><td>miRNA 3' agUCA--AAGGAGAC--------GUUUGUCAa 5'<br>|||  |  |||||        ||:|||||<br>Target 5' aaAGTAATGGCTCTGGGTTGGGCCAGACAGTt 3'</td><td>5137 - 5168</td><td>136.00</td><td>-14.90</td></tr>
</table>

**Figure S2: miR-452-5p and SMAD4 mRNA duplex showing pivotal binding sites predicted by miRanda (screenshot), related to Figure 6.**

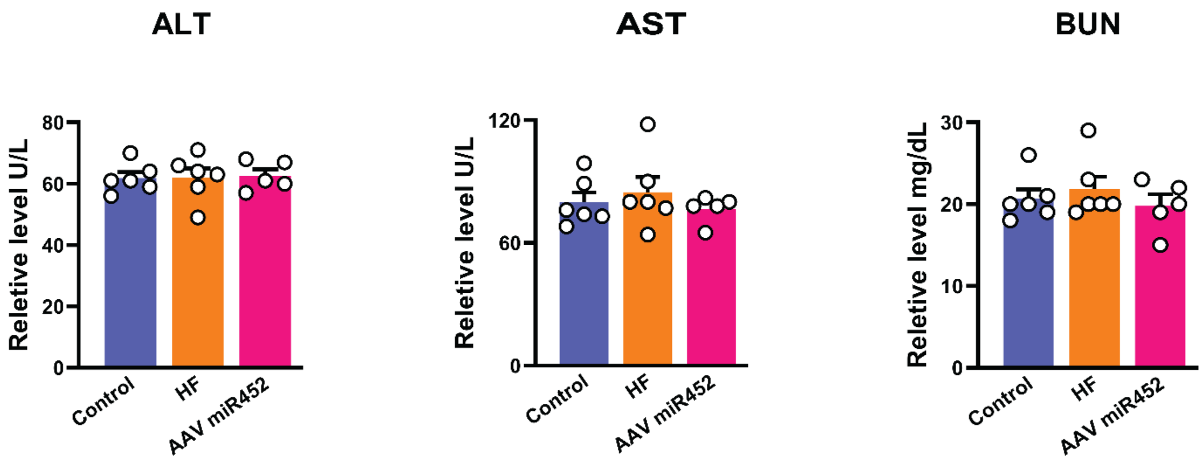


**Figure S3. Serum ALT, AST, and BUN levels in heart failure (HF) and HF treated with AAV miR452 compared to control, related to Figure 8.**

Data are presented as mean ± SEM. One-way ANOVA showed no significant difference of ALT, AST, and, BUN levels between the control, HF, and HF treated with AAV miR452 groups, n = 6 independent experiments. ALT; Alanine transaminase, AST: aspartate aminotransferase, BUN; blood urea nitrogen.

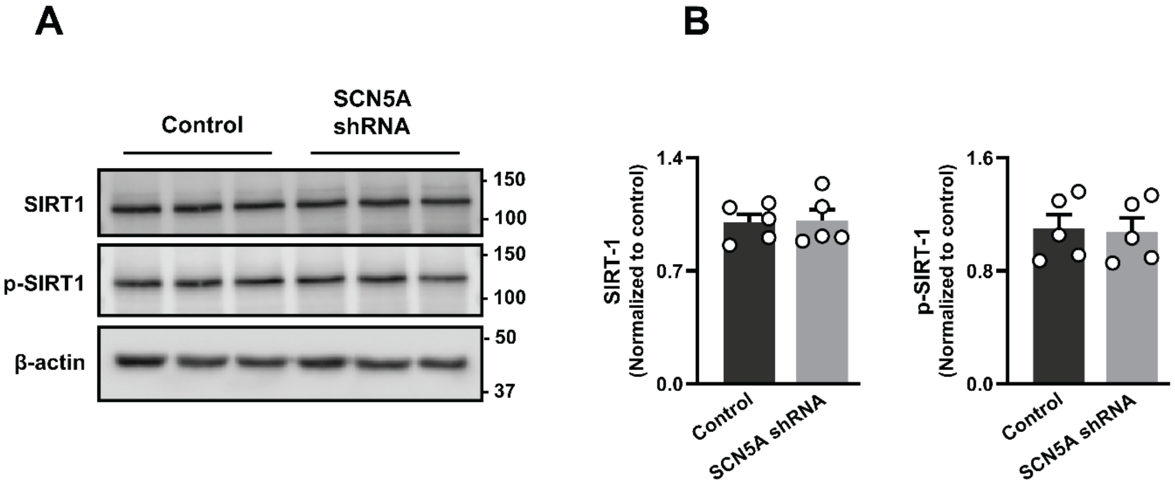


**Figure S4. SIRT1 expression in SCN5A knockdown HCF, related to Figure 6.**

**A)** Representative immunoblots of SIRT1 and phosph-SURT1 expression **B)** Quantitative analysis presenting the unaltered expression of SIRT-1 and phospho-SIRT-1 after SCN5A knockdown compared to control. Data are expressed as mean ± SEM, paired t-test showed that SIRT1 expression was non-significant in SCN5A knockdown HCF than control, n=5 independent experiments.
